# The major outer sheath protein forms distinct conformers and multimeric complexes in the outer membrane and periplasm of *Treponema denticola*

**DOI:** 10.1038/s41598-017-13550-6

**Published:** 2017-10-16

**Authors:** Robbins Puthenveetil, Sanjiv Kumar, Melissa J. Caimano, Abhishek Dey, Arvind Anand, Olga Vinogradova, Justin D. Radolf

**Affiliations:** 1 Department of Molecular and Cell Biology, University of Connecticut, 91 North Eagleville Road, Storrs, CT USA; 2 Department of Medicine, UConn Health, 263 Farmington Avenue, Farmington, CT USA; 3 Department of Pediatrics, UConn Health, 263 Farmington Avenue, Farmington, CT USA; 4 Department of Molecular Biology and Biophysics, UConn Health, 263 Farmington Avenue, Farmington, CT USA; 5 Department of Pharmaceutical Sciences, University of Connecticut, 69 North Eagleville Road, Storrs, CT USA; 6 Department of Genetics and Genome Science, UConn Health, 400 Farmington Avenue, Farmington, CT USA; 7 Department of Immunology, UConn Health, 263 Farmington Avenue, Farmington, CT USA

## Abstract

The major outer sheath protein (MOSP) is a prominent constituent of the cell envelope of *Treponema denticola* (TDE) and one of its principal virulence determinants. Bioinformatics predicts that MOSP consists of N- and C-terminal domains, MOSP^N^ and MOSP^C^. Biophysical analysis of constructs refolded *in vitro* demonstrated that MOSP^C^, previously shown to possess porin activity, forms amphiphilic trimers, while MOSP^N^ forms an extended hydrophilic monomer. In TDE and *E. coli* expressing MOSP with a PelB signal sequence (PelB-MOSP), MOSP^C^ is OM-embedded and surface-exposed, while MOSP^N^ resides in the periplasm. Immunofluorescence assay, surface proteolysis, and novel cell fractionation schemes revealed that MOSP in TDE exists as outer membrane (OM) and periplasmic trimeric conformers; PelB-MOSP, in contrast, formed only OM-MOSP trimers. Although both conformers form hetero-oligomeric complexes in TDE, only OM-MOSP associates with dentilisin. Mass spectrometry (MS) indicated that OM-MOSP interacts with proteins in addition to dentilisin, most notably, oligopeptide-binding proteins (OBPs) and the β-barrel of BamA. MS also identified candidate partners for periplasmic MOSP, including TDE1658, a spirochete-specific SurA/PrsA ortholog. Collectively, our data suggest that MOSP destined for the TDE OM follows the canonical BAM pathway, while formation of a stable periplasmic conformer involves an export-related, folding pathway not present in *E. coli*.

## Introduction

Periodontitis, the most common cause of tooth loss worldwide, is a chronic inflammatory condition of the periodontium^1,2^. Periodontitis results from the interplay between the overgrowth of microorganisms, the host inflammatory response, and genetic and environmental factors^1,2^. Molecular phylogenetics has revealed hundreds of bacterial species in subgingival plaque, including ~50 treponemal phylotypes^3,4^. Among the latter, *Treponema denticola* is the most abundant and the best characterized^5–7^. Along with *Porphyromonas gingivalis* and *Tannerella forsythia, T. denticola* forms the “Red Complex” that is strongly associated with severity and progression of periodontal disease^8^.

The 53-kDa major outer sheath protein, MOSP (TDE0405/NP_971019.1), is one of the most abundant polypeptides in *T. denticola* and a principal virulence determinant^6,7^. In addition to forming large water-filled channels in the outer membrane (OM)^9–11^, MOSP has myriad pathogenesis-related biological activities^11–22^, while also serving as a partner for the dentilisin protease complex in the *T. denticola* OM^11,12,23^ (see below). When the sequence of MOSP was reported^15^, it was presumed that the entire polypeptide forms an OM-spanning β-barrel. However, since then, evidence has emerged indicating that MOSP and its orthologs in the pathogenic treponemes (the *T. pallidum* repeat protein [Tpr] family) possess a bipartite architecture consisting of conserved N- and C-terminal domains (MOSP^N^ and MOSP^C^), with MOSP^C^ forming the OM-embedded β-barrel^10,24,25^. Moreover, native MOSP appears to exist as two distinct conformers in the OM and periplasm^10,26^.

Fundamental to the progression of periodontitis is the ability of its etiologic agents to penetrate and degrade periodontal tissue^1,2^. *T. denticola* contains several proteases^6,7^ that may facilitate this process. Two trypsin-like oligopeptidase B proteases, TDE2140/NP_972741.1 and TDE1195/NP_971802.1, have been identified; TDE2140 has been shown to cleave C-terminal to Arg residues^27^, while TDE1195 is postulated to be a lysine-specific protease^28^. Dentilisin, a chymotrypsin-like protease, is believed to promote bacterial penetration of epithelial cells by digesting tight junctional and extracellular matrix proteins^6,7^. Dentilisin also cleaves Factor H bound to the surface of *T. denticola* via the lipoprotein FhbB, theoretically dysregulating complement activation in the subgingival crevice and promoting bacterial overgrowth, host cell death, and abscess formation^29,30^. Dentilisin is a multimeric lipoprotein complex formed by the proteins PrtP, PrcA1, PrcA2 and PrcB^23,31–36^. PrtP hydrolyzes PrcA to produce PrcA1 and PrcA2, all three of which remain tightly associated^33^. Strains lacking PrcB fail to produce PrtP and, correspondingly, express full-length PrcA and exhibit no proteolytic activity^35^.

In archetypal dual membrane (diderm) bacteria (*e.g., Escherichia coli*), proteins destined for the OM exist in the periplasm only as unfolded intermediates^37^. In this report, we confirmed our previous findings^10^ that MOSP represents an unprecedented exception to this “one compartment” paradigm for exported bacterial proteins. We demonstrate herein that MOSP not only forms physically distinct, stable OM and periplasmic trimeric conformers but that formation of the latter involves an export-related folding pathway that may be spirochete-specific. Both conformers serve as platforms for complex formation, however, the complexes are different; only the OM trimer associates with dentilisin. Moreover, mass spectrometric (MS) analysis of the OM conformer complex revealed that MOSP interacts with a broader repertoire of proteins than just dentilisin, most notably, OppA substrate-binding proteins (SBPs), suggesting a possible surface to cytoplasm conduit for peptide nutrients generated on the bacterial surface by proteases, including dentilisin. MS also identified several candidate partners for the periplasmic MOSP complex, including TDE1658, a spirochete-specific SurA/PrsA ortholog and possible holdase/foldase functional chimera. We hypothesize that interactions with these candidates divert newly exported MOSP from the OM biogenesis pathway, promoting stabilization of the alternative periplasmic conformation.

## Results

### MOSP^C^ forms an amphiphilic β-barrel, while MOSP^N^ forms a hydrophilic, extended structure

**Figure 1:**
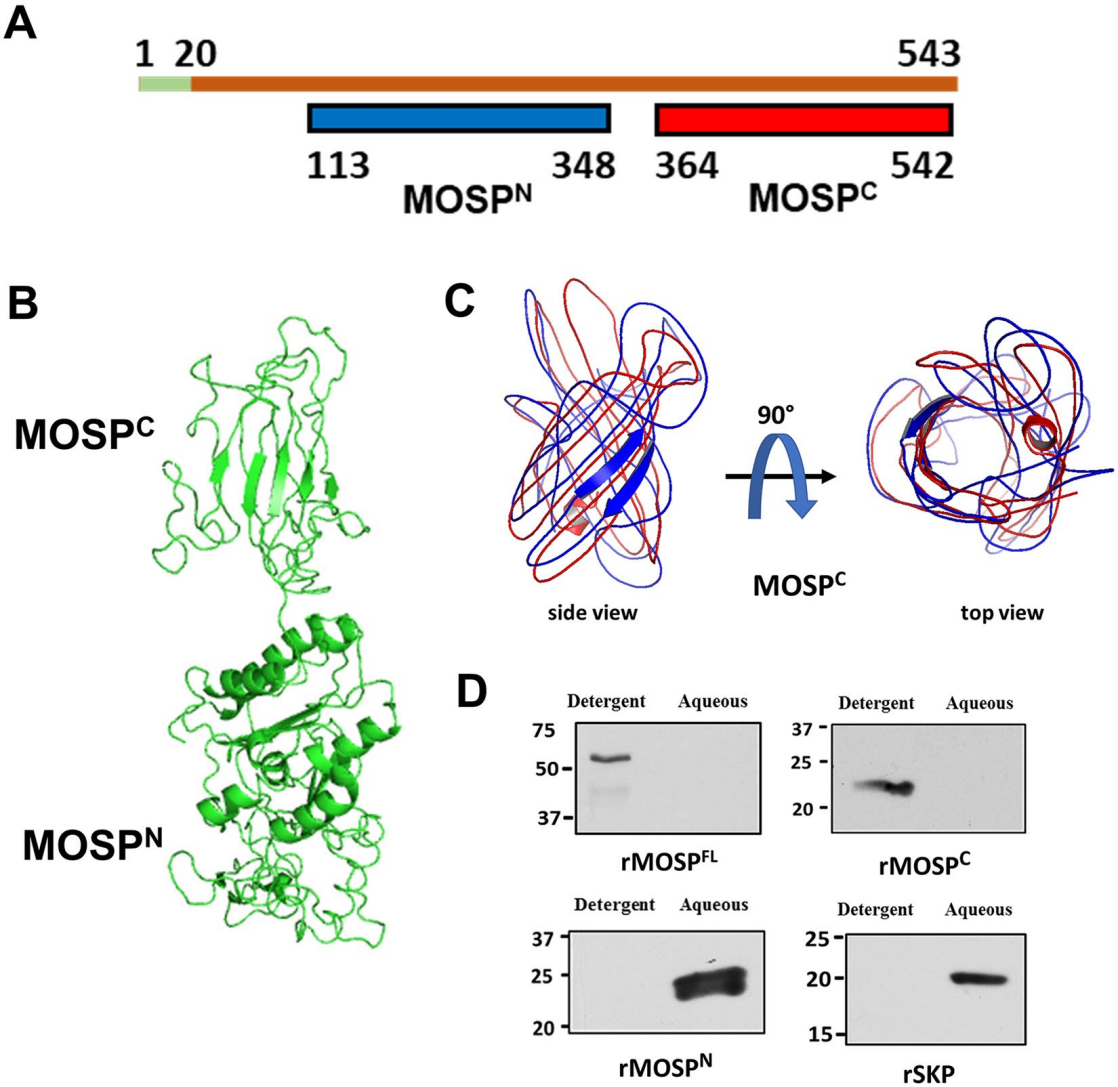
MOSP^Fl^ contains N- and C-terminal domains with different solubility properties. **(A)** Domain architecture of MOSP^Fl^ predicted by InterProScan. The first 20 amino acids (shown in green) contain the cleaved signal sequence. The portion of MOSP^Fl^ colored in blue denotes the N-terminal domain, MOSP^N^, while red denotes the C-terminal domain, MOSP^C^. **(B)** Optimal model for MOSP^Fl^, generated by I-TASSER, predicting a bipartite architecture in which MOSP^N^, contains substantial α-helical content and MOSP^C^ is predominantly β-sheet. **(C)** Overlay of models generated by I-TASSER predicting that MOSP^C^ is a 10-stranded β-barrel. (**D**) 10 µg of recombinant MOSP^Fl^, MOSP^C^, MOSP^N^ and Skp were phase-partitioned in TX-114 and separated on SDS-PAGE. Lanes show detergent-enriched and aqueous phases probed with antisera directed against each recombinant protein. Panel D presents cropped images; the full-length images are presented in Supplementary Figure 4.

Sequence analysis with InterProScan^38^ and Pfam^39^ predicts that full-length MOSP (MOSP^Fl^) has a bipartite architecture consisting of N- and C-terminal domains, MOSP^N^ and MOSP^C^, respectively (Fig. 1A). BOCTOPUS^40^, a trans-membrane β-barrel prediction tool, shows that MOSP^C^ has a higher propensity to form a β-barrel. Five models with C-scores ranging from −1.73 to −2.4 were generated for MOSP^Fl^ using the Iterative Threading ASSEmbly Refinement (I-TASSER) software. The above sequence-based domain predictions, along with the confidence scores, were used to select the optimal model for MOSP^Fl^ (Fig. 1B). This model predicts that MOSP^N^ possesses substantial α-helical content, while MOSP^C^ is predominantly β-stranded, in agreement with secondary structure analyses for the two refolded domains previously obtained by CD spectroscopy^10^. Further modeling of MOSP^C^ by I-TASSER yielded a 10-stranded β-barrel that aligned best to structures of OpcA (PDB id:2VDF) and OmpT (PDB id:1I78) (Fig. 1C). We next used Triton X-114 (TX-114) phase partitioning to compare the physical properties of recombinant MOSP^Fl^, MOSP^C^, and MOSP^N^. MOSP^Fl^ and MOSP^C^ partitioned into the detergent-enriched phase, while MOSP^N^ and Skp (TDE2602/NP_973200.1), a periplasmic chaperone^10,37^, were recovered in the aqueous phase (Fig. 1D). These results collectively point to MOSP^C^ as the OM-embedded region of MOSP^Fl^ ^10^.

**Figure 2:**
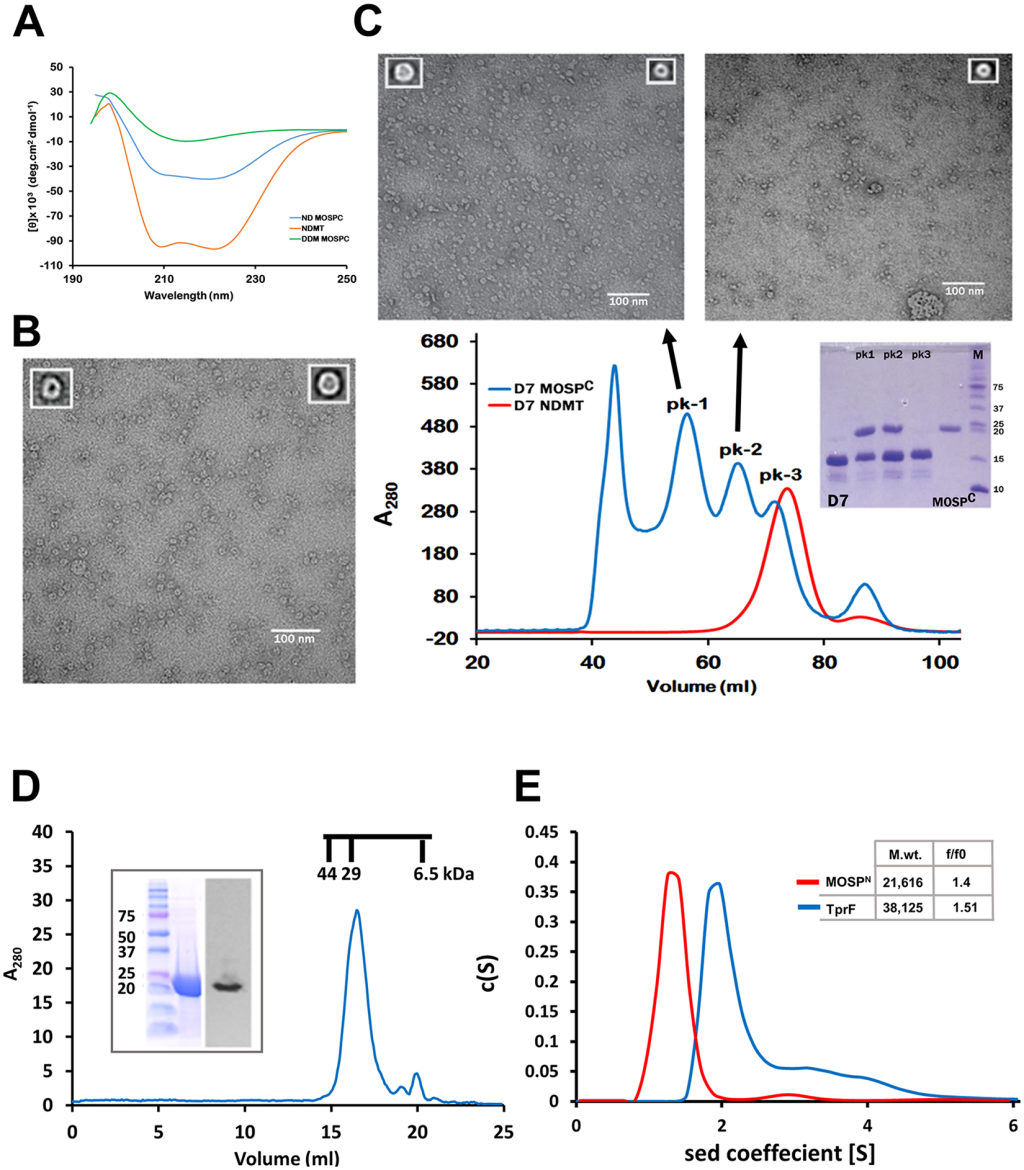
MOSP^C^ forms trimers capable of inserting into bilayer nanodics while MOSP^N^ forms an extended hydrophilic structure. (**A)** CD spectra of MOSP^C^ refolded in 1% DDM (green), empty E3D1 nanodiscs (orange), and MOSP^C^ encapsulated in E3D1 discs (blue). **(B)** Negatively-stained TEM image of MOSP^C^ in E3D1 discs with representative averaged images of an inserted monomer and trimer shown as insets in upper left and right, respectively. **(C)** Chromatogram of MOSP^C^ incorporated into small D7 discs separated by SEC on a S200 column (blue). The chromatogram in red represents empty discs run separately as a control. The SDS-PAGE gel above the chromatogram shows the contents of peaks 1 through 3; lanes 1 and 5 contain D7 and MOSP^C^ for reference, while lane 6 contains molecular mass markers (kDa). Above the chromatograms are TEM images of peaks 1 and 2 with insets containing representative averaged images. Peak 1 contains trimers and monomers, while peak 2 contains only monomers. **(D)** SEC of refolded recombinant MOSP^N^ with SDS-PAGE and immunoblot shown as inset. Chromatographic molecular weight standards are shown above the major peak containing MOSP^N^. **(E)** Sedimentation velocity experiments performed on MOSP^N^ and TprF using AUC. The inset shows the buoyant molecular masses and frictional coefficient ratios (f/fo) as determined through c(S) analysis using SEDFIT.

To extend these findings, we examined the refolding of MOSP^C^ in n-Dodecyl-β-D-Maltoside (DDM) and its further incorporation into nanodiscs, a true lipidic environment for integral membrane proteins^41,42^. CD spectroscopy for detergent-refolded MOSP^C^ gave a broad minimum centering on 218 nm, indicating a preponderance of β-strands, whereas empty MSP1E3D1 (E3D1) scaffolds exhibited their signature α-helical minima at 208 and 222 nm (Fig. 2A). As expected, MOSP^C^ incorporated into E3D1 discs gave an averaged spectrum arising from the presence of both α and β secondary structural elements (Fig. 2A). The representative averaged transmission electron microscopy (TEM) images for MOSP^C^ clearly showed the presence of trimers composed of closed, circular monomers with well-demarcated central channels (Fig. 2B), consistent with studies showing that the porin-like function of MOSP resides within MOSP^C^ ^10^. Along with trimers, some monomers also were observed (Fig. 2B), raising the possibility that trimerization could have resulted from the entrapment of multiple monomers within the space provided by the large E3D1 discs^43^. To resolve this, we employed small nanodiscs^44^ because of their more restrictive capacity to accommodate trimers. The size exclusion chromatogram obtained for MOSP^C^-D7 nanodiscs consisted of three peaks (Fig. 2C). SDS-PAGE of peaks 1 and 2 confirmed the presence of MOSP^C^-incorporated discs; peak 3 contained empty D7 discs. TEM analysis of peak 2 revealed monomers, while peak 1 contained a mixture of monomers and trimers (Fig. 2C). Size exclusion chromatography (SEC) of refolded MOSP^N^ yielded one major peak comparable in size to the MOSP^N^ monomer (Fig. 2D). Analytical ultracentrifugation (AUC) yielded a similar estimated mass (Fig. 2E). In addition, the f/fo ratio of MOSP^N^ (1.4) obtained by AUC closely matched the value (1.5) obtained for TprF (Fig. 2E), a truncated Tpr protein previously shown by small angle X-ray scattering to form an extended structure^25^. Taken together, these results not only support the bipartite model but also indicate that MOSP^C^ is sufficient for trimerization of native MOSP^10,11^.

### Native MOSP is bipartite and exists as OM-embedded and periplasmic trimmers

**Figure 3:**
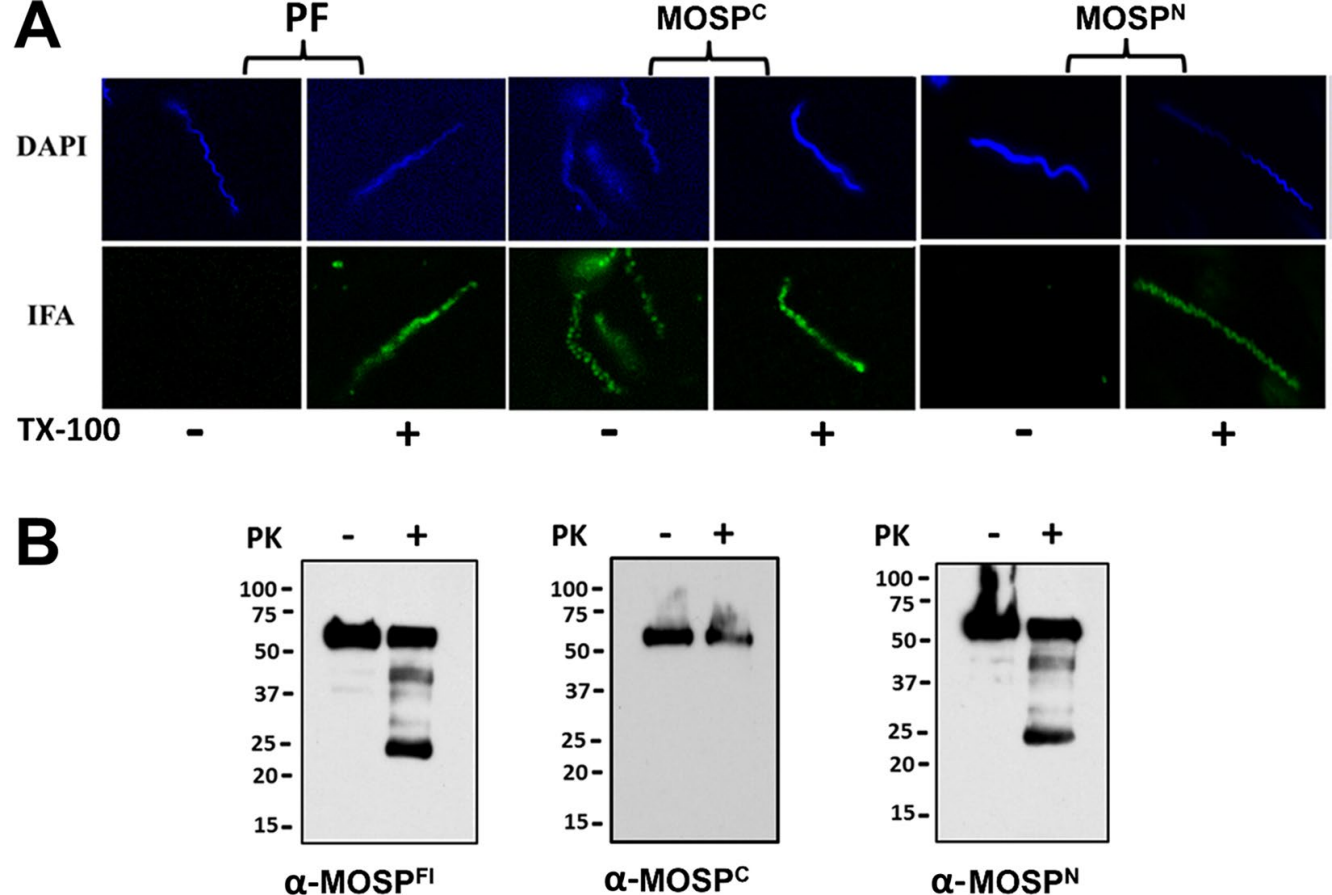
Immunolabeling and surface proteolysis of *T. denticola* confirm bipartite architecture of native MOSP and the presence of OM and periplasmic conformers. (**A**) Immunolabeling of *T. denticola* encapsulated in gel microdroplets in the absence (−) or presence (+) of 0.05% TX-100. Organisms were probed with antibodies directed against MOSP^C^, MOSP^N^, and periplasmic flagella (PF). (**B**) Surface proteolysis of *T. denticola* exposed to proteinase K (PK) for 1 hour. Immunoblot analysis of MOSP before (−) and after (+) treatment with PK using antisera directed against MOSP^Fl^, MOSP^C^ and MOSP^N^. Molecular mass standards (kDa) are indicated on the left of each gel.

We next conducted experiments to localize MOSP^N^ and MOSP^C^ in *T. denticola*. First, we performed indirect immunofluorescence analysis (IFA) of treponemes encapsulated in gel microdroplets; this methodology not only preserves the integrity of the fragile *T. denticola* OM but allows for its controlled removal using low detergent concentrations (here 0.05% Triton X-100)^10,26,45^. Labeling of intact organisms was observed only for MOSP^C^ (Fig. 3A). In contrast, following detergent treatment, both MOSP^C^ and MOSP^N^ were labeled, as were periplasmic flagella. These results are consistent with the presence of an OM-associated form of MOSP^Fl^, in which only MOSP^C^ is surface-exposed, and a full-length periplasmic conformer^10,26^. To support this interpretation, we examined the susceptibility of native MOSP to proteinase K (PK) digestion. If MOSP^Fl^ exists as bipartite OM-embedded and periplasmic conformers, then PK-treatment of motile *T. denticola* should yield MOSP^Fl^ (~52 kDa), a proteolytic degradation product corresponding to MOSP^N^ (~25 kDa), and little to no MOSP^C^. Indeed, this is what we observed (Fig. 3B). While the presence of a strong band for MOSP^Fl^ indicates that substantial amounts of the protein were inaccessible to PK treatment, a decrease in MOSP^Fl^ also was evident (Fig. 3B).

**Figure 4:**
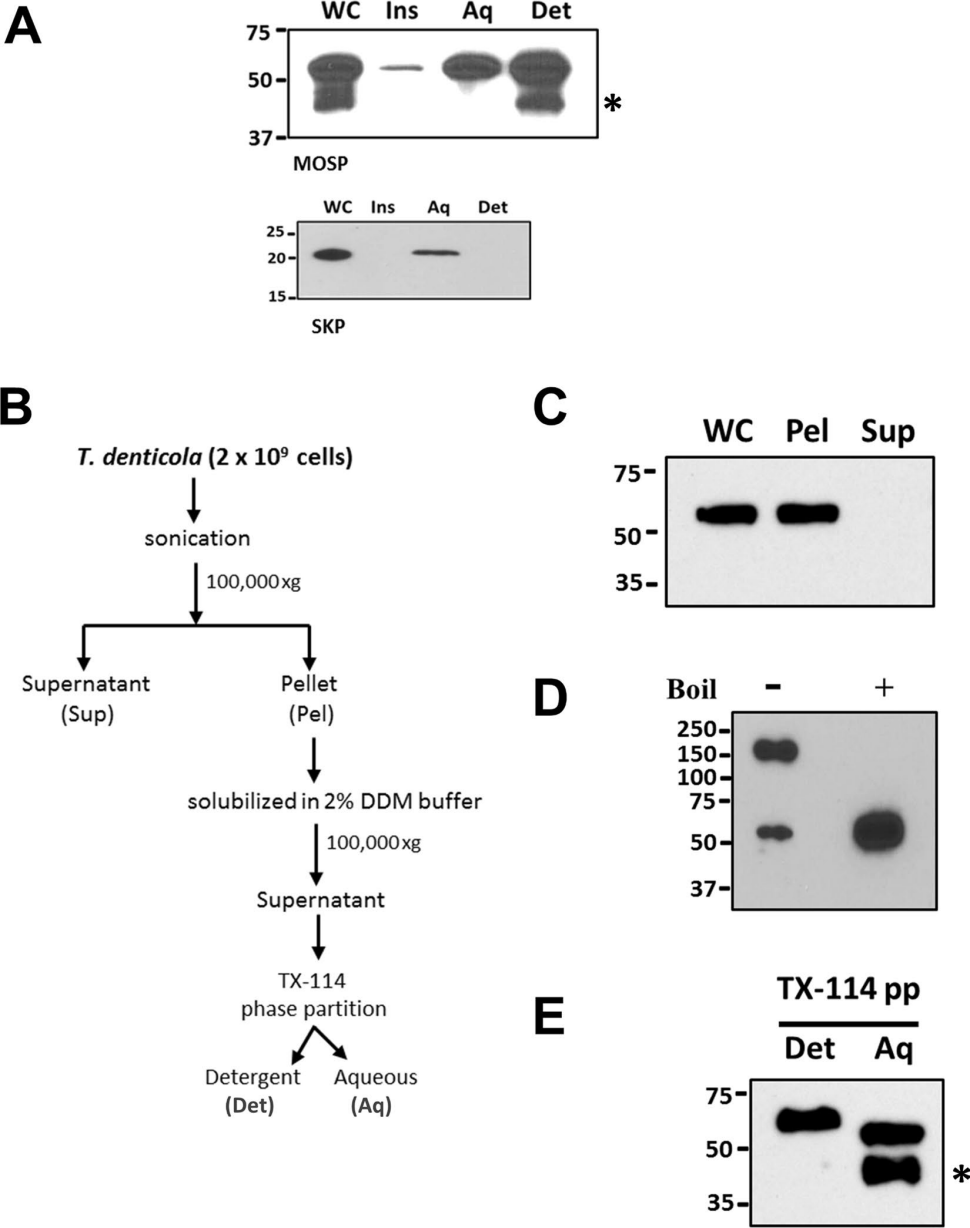
OM and periplasmic conformers in *T. denticola* are trimeric. (**A)** Upper panel: detergent-enriched (Det) and aqueous (Aq) phases following TX-114 phase partitioning of *T*. *denticola*, SDS-PAGE, and immunoblotting with MOSP^Fl^ antiserum; also shown are whole cells (WC) and TX-114-insoluble material (Ins). Lower panel: TX-114 phase partitioning of the periplasmic chaperone Skp in *T. denticola*. (**B)** Protocol delineating fractionation of *T. denticola* lysate using DDM followed by TX-114 phase partitioning. **(C)** Immunoblot analysis of the pellet (Pel) and supernatant (Sup) following ultracentrifugation of sonicated *T. denticola* whole cells (WC). **(D)** SDS-PAGE and immunoblot analysis (using anti-MOSP^Fl^) of DDM-solubilized pellet from the *T. denticola* lysate. Samples were run with (+) and without (−) boiling. **(E)** TX-114 phase partitioning of DDM-solubilized material. Asterisks in panels A and E indicate a degradation product of MOSP^Fl^ (52-kDa). Molecular mass standards (kDa) are indicated on the left of each gel.

If native MOSP exists as both OM-embedded and periplasmic forms, one would expect that the two could be separated by TX-114 phase partitioning. Figure 4A shows that comparable amounts of MOSP^Fl^ were recovered in both detergent-enriched and aqueous phases, while native periplasmic Skp, like its recombinant counterpart, was recovered exclusively in the aqueous phase. We then devised a novel fractionation scheme employing DDM solubilization of pelleted *T. denticola* sonicate followed by TX-114 phase partitioning (Fig. 4B) to demonstrate the presence of distinct conformers. Analysis of the DDM-solubilized material without boiling revealed that a large majority of MOSP migrated with a molecular mass of ~150 kDa, consistent with a trimer, while boiling yielded only monomers (Fig. 4D). If the DDM-solubilized material was subjected to phase partitioning prior to SDS-PAGE, approximately equal amounts of MOSP were recovered in both phases (Fig. 4E). Collectively, these results indicate that both MOSP conformers exist predominantly as trimers and that the periplasmic trimer associates with the protoplasmic cylinder.

### PelB-MOSP is bipartite, trimeric and localizes exclusively to the OM

**Figure 5:**
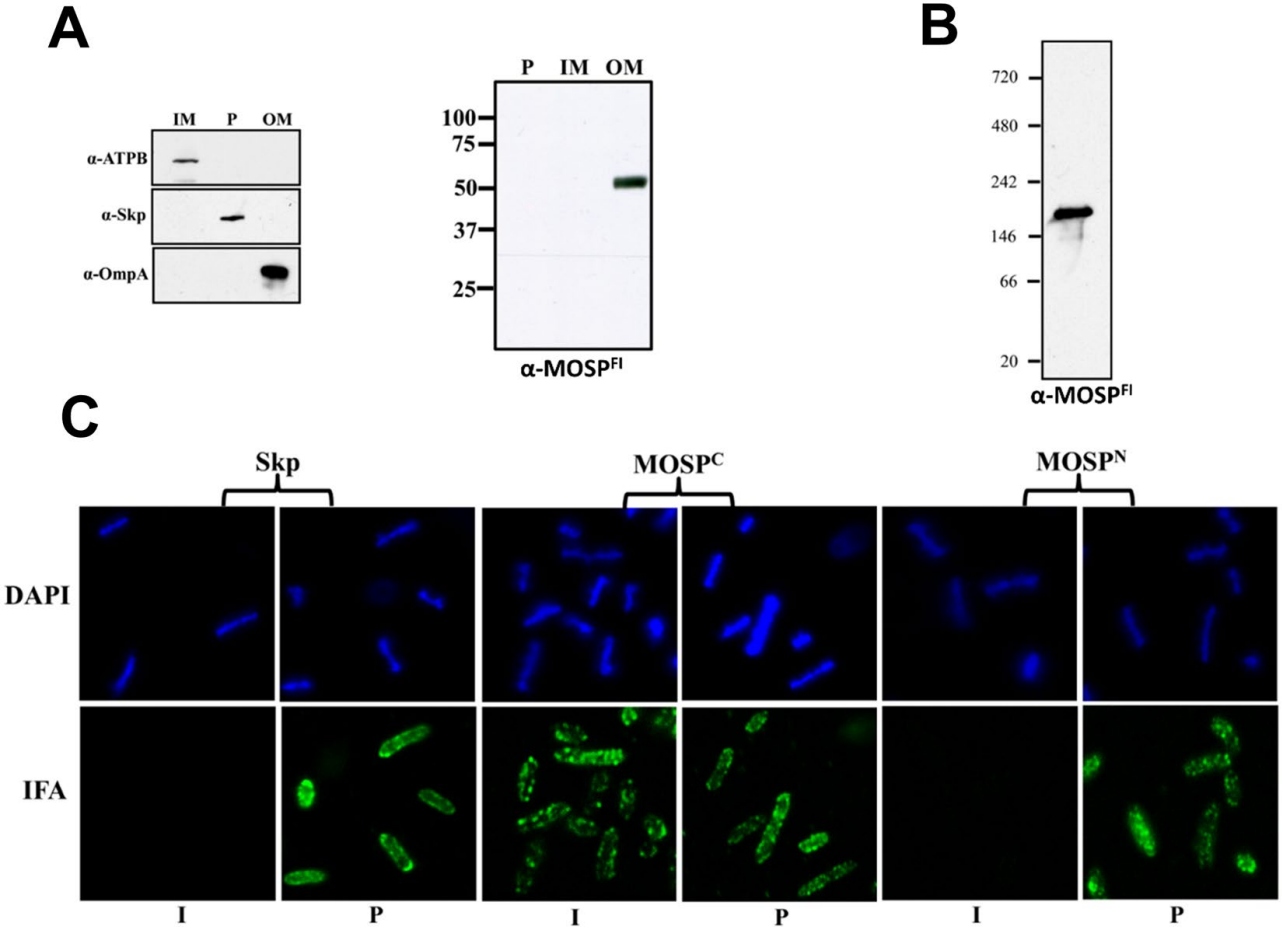
MOSP expressed in *E. coli* with a PelB signal sequence is bipartite and exclusively OM-localized. (**A)** Inner membrane (IM), periplasmic (P), and outer membrane (OM) fractions from *E. coli* C41 (DE3) expressing MOSP with PelB signal sequence were separated by SDS-PAGE and immunoblotted against MOSP^Fl^ antiserum. Antisera against ATPB, Skp and OmpA served as markers for the IM, P and OM fractions, respectively. **(B)** BN-PAGE of DDM-solubilized MOSP^Fl^ from the OM fraction. **(C)** IFA of intact (I) or permeabilized (P) *E. coli* C41 (DE3) expressing OM-localized MOSP^Fl^ probed with rat antisera against MOSP^C^, MOSP^N^, and Skp. Antibody binding was detected with 1 μg/ml goat anti-rat Alexa Fluor 488 conjugate.

We next asked whether the formation of OM and periplasmic conformers would occur in diderms other than *T. denticola*. As shown in Fig. 5A, MOSP expressed in *E. coli* with a PelB leader (PelB-MOSP) fractionated with the OMs. Moreover, following DDM solubilization of the OM, PelB-MOSP was trimeric (Fig. 5B). We then used IFA to examine the domain topology of PelB-MOSP. Intact *E. coli* expressing PelB-MOSP labeled with antibodies to MOSP^C^, but not MOSP^N^, while MOSP^N^ and the Skp periplasmic control were detected only in permeabilized organisms (Fig. 5C).

### Both MOSP conformers form multimeric complexes but only the OM conformer associates with dentilisin

**Figure 6:**
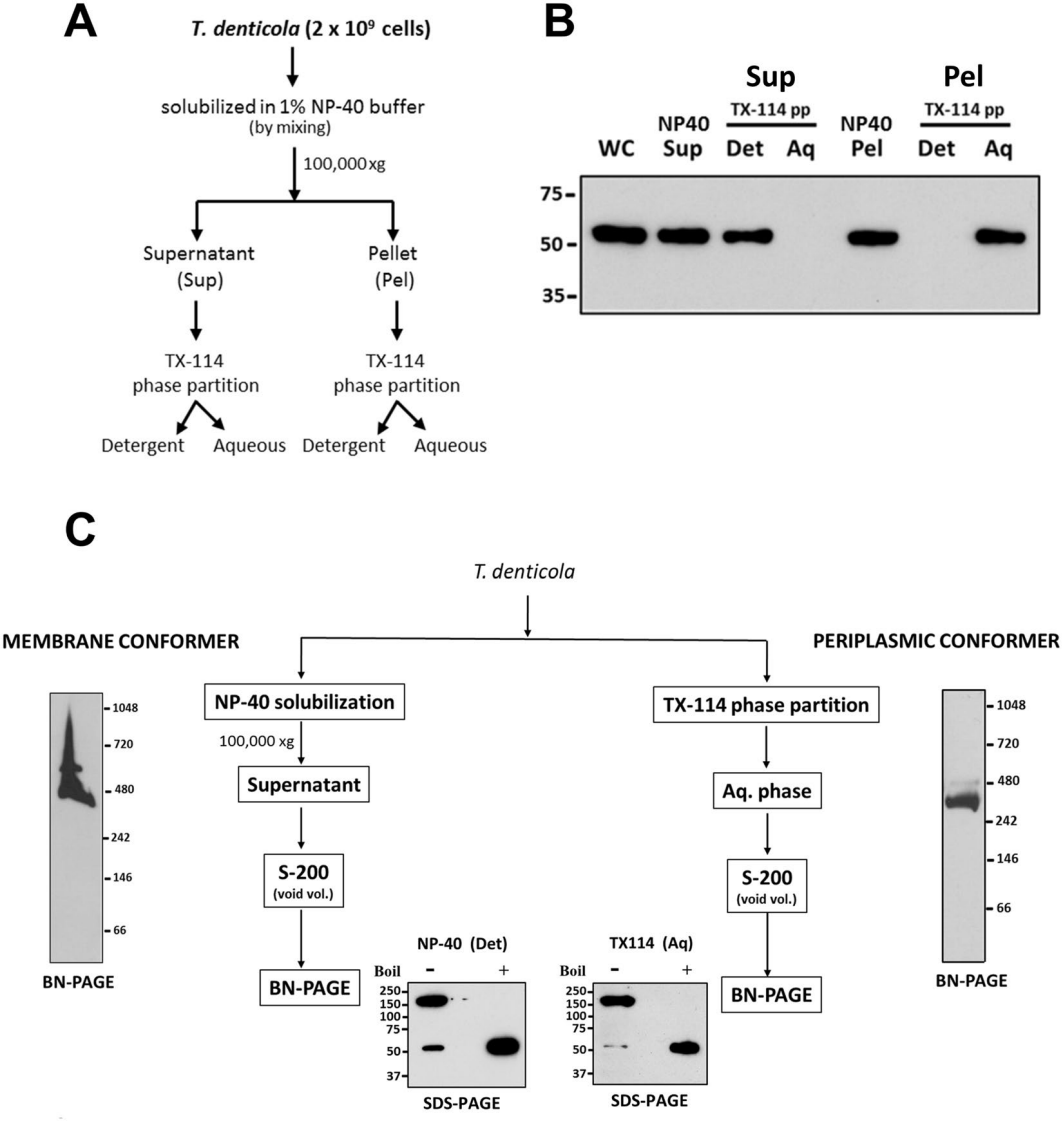
OM- and periplasmic-MOSP form SDS-stable trimers and distinct multimeric complexes. (**A)** Protocol outlining the use of NP-40 to separate OM and periplasmic conformers. **(B)** Immunoblot of the NP-40 soluble (supernatant; NP-40 Sup) and insoluble (pellet; NP40 Pel) fractions obtained by ultracentrifugation which were then separately subjected to TX-114 phase partitioning (TX-114 pp). **(C)** Immunoblot of BN-PAGE gels of NP-40 supernatant and TX-114 aqueous phase using anti-MOSP^Fl^ antiserum. Also shown are SDS-PAGE gels of the separated conformers without (−) and with (+) boiling followed by immunoblot analysis with anti-MOSP^Fl^. Molecular mass standards (kDa) are indicated on the left (SDS-PAGE) or right (BN-PAGE) of each gel.

Native MOSP trimers interact with dentilisin^11,12,23^. We next sought to ascertain whether this interaction is exclusive to one of the conformers. While the TX-114 aqueous phase is amenable to further manipulations, the detergent-enriched phase is difficult to work with because the micelles form aggregates that interfere with analytical techniques performed under non-denaturing conditions, even at 4 °C (well below the 20 °C cloud point of TX-114^46^). We discovered that it was possible to circumvent this problem using Nonidet P-40 (NP-40) (Fig. 6A). As shown in Fig. 6B, strong MOSP immunoreactivity was detected in both the NP-40 supernatant and pellet. Subsequent TX-114 phase partitioning revealed that the NP-40 solubilized MOSP partitioned exclusively into the detergent-enriched phase, while the NP-40 insoluble MOSP partitioned exclusively into the aqueous phase (Fig. 6B). These results established that NP-40-solubilized MOSP was the OM conformer (OM-MOSP). Immunoblotting of OM and periplasmic conformers separated by BN-PAGE revealed that both exist as higher molecular weight complexes with approximate molecular masses of ~480- and 400-kDa, respectively (Fig. 6C). Unlike the periplasmic conformer (periplasmic-MOSP), OM-MOSP also formed a larger, less abundant complex with a molecular mass of ~600 kDa. Examination of the two conformers by SDS-PAGE without boiling revealed that, as seen earlier (Fig. 4D), both consisted predominantly of 150 kDa SDS-stable trimers (Fig. 6C).

**Figure 7:**
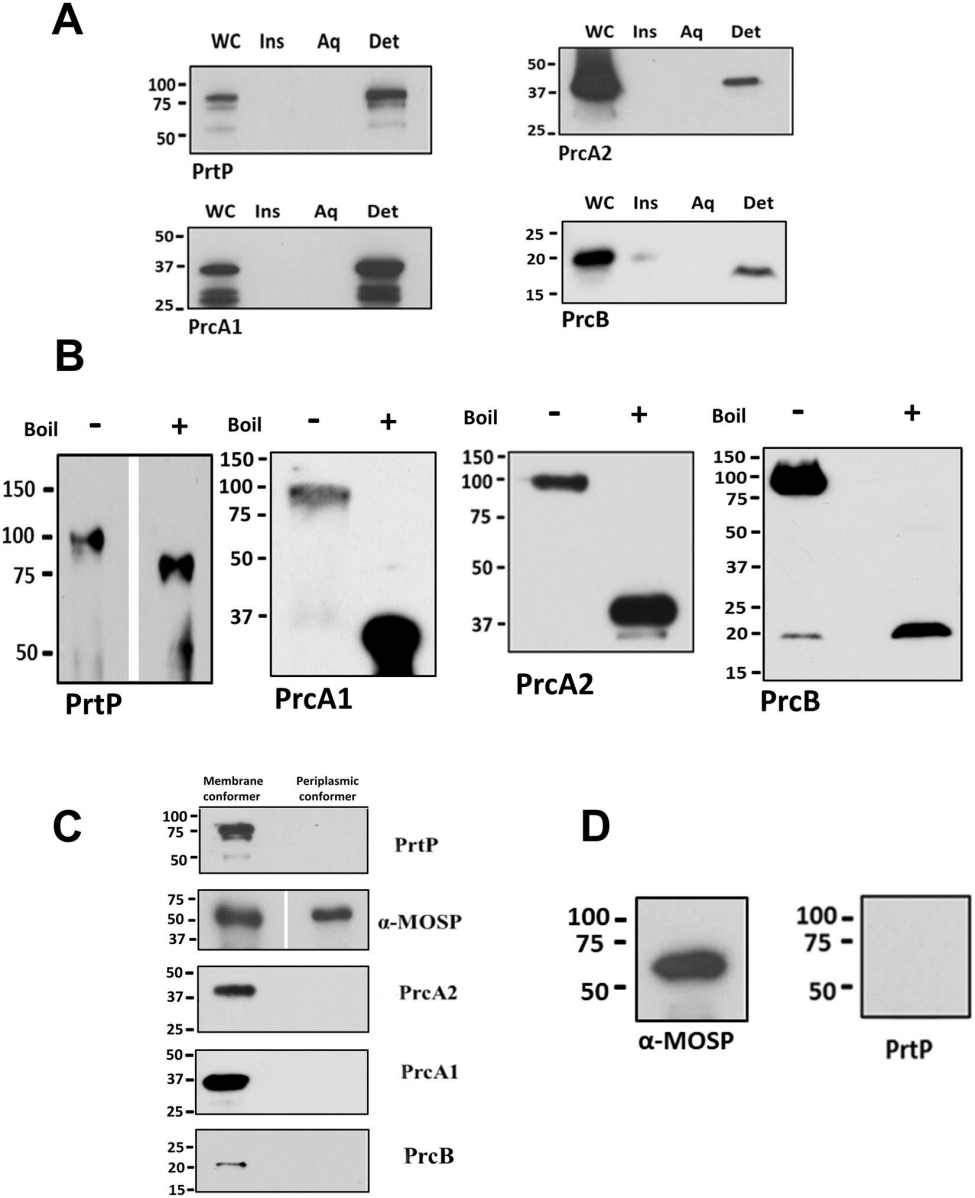
The dentilisin complex associates only with OM-MOSP. (**A)** SDS-PAGE and immunoblot analysis of TX-114 phase partitioned *T*. *denticola* cells using antisera directed against PrtP, PrcA1, PrcA2, and PrcB. Lanes: whole cells (WC), TX-114 insoluble material (Ins), aqueous (Aq) and detergent (Det) phase. **(B)** SDS-PAGE gels of the NP-40 supernatant without (−) or with (+) boiling followed by immunoblot analysis using the same antisera as in Panel A. **(C)** Eluates from Co-IP of OM and periplasmic conformers were immunoblotted with antisera against the four dentilisin components. **(D)** SDS-PAGE and immunoblot analysis of eluate when 2% DDM was added to the NP-40 supernatant prior to Co-IP. Molecular mass standards (kDa) are indicated on the left of each gel.

For many years, dentilisin was defined as an SDS-stable complex composed of PrtP, PrcA1, and PrcA2^31–34^. Recently, Godovikova and co-workers^23,35^ identified a fourth component, PrcB, encoded by the first gene in the same operon as *prtP* and *prcA*. As noted previously^23,35^, all four partition exclusively into the TX-114 detergent phase (Fig. 7A). Immunoblotting of the unboiled NP-40 supernatant confirmed the presence of PrtP, PrcA1, PrcA2, and PrcB in an SDS-stable ~100 kDa complex (Fig. 7B). In co-immunoprecipitation (Co-IP) experiments using MOSP^Fl^ antiserum, MOSP was pulled down from both the NP-40 supernatant and TX-114 aqueous phase, but dentilisin components were detected only in the former (Fig. 7C). When 2% DDM was added to the NP-40 supernatant prior to Co-IP, PrtP was not pulled down (Fig. 7D), indicating dissociation of MOSP from dentilisin.

### Identification of candidate MOSP interacting partners

We performed MS to determine whether OM-MOSP partners with proteins other than dentilisin and to identify components of the periplasmic-MOSP complex. Two complementary approaches were employed. In one, we performed Co-IPs on the NP-40 supernatant (containing OM-MOSP) and the TX-114 aqueous phase (containing periplasmic MOSP) with agarose crosslinked MOSP^Fl^ antiserum and analyzed the two eluates (Co-IP_Det and Co-IP_Aq, respectively). In the other, we fractionated the NP-40 supernatant and TX-114 aqueous phase by BN-PAGE and excised the ~480- (the predominant MOSP-containing band) and ~400-kDa MOSP bands (Band_Det and Band_Aq, respectively). For each dataset, we filtered out proteins with expectation values <1 × 10^−6^ and used SignalP and LipoP to identify proteins predicted to contain a cleaved N-terminal signal peptide, a minimum requirement for interaction with either MOSP conformer. Suppl. Table 1–4 contains the complete raw MS datasets.

MS analysis revealed 49 candidate partners in the Co-IP_Det, including 31 putative lipoproteins and 16 proteins with annotations (Table 1). PrcA, represented by peptides from both PrcA1 and PrcA2 (Suppl. Figure 1), was the only dentilisin component identified. The *T. denticola* reference strain 35405 genome is missing PrtP due to a sequencing error^36^; however, manual interrogation of the MS datasets also failed to identify PrtP peptides. Among the remaining 15 annotated proteins, six are predicted substrate-binding proteins (SBPs), four of which are oligopeptide-binding proteins (OBPs). One of the four, TDE1072, is annotated simply as a lipoprotein. However, CDD and Pfam both identified the signature SBP family 5 domains found in OppAs, while the locus containing the *tde1072* gene also encodes contiguous permeases (TDE1073-74) and nucleotide binding domains (TDE1075-76), thereby forming a complete Opp transporter. Two proteins, TDE2055 and TDE2056, annotated as “hemin-binding protein B” and “outer membrane hemin-binding protein A”, are probably not involved in heme uptake. By CDD and BLAST search, both contain ZinT domains, suggesting that they function as auxiliary proteins for zinc uptake by the Znu ABC transporter^47^. TDE2257, annotated as “5′-nucleotidase”, is a putative NadN, a periplasmic enzyme that processes exogenously acquired nicotinamide adenine dinucleotide to adenosine and nicotinamide ribonucleotide^48^. Of the non-lipoproteins in the Co-IP_Det, TDE2601/BamA was particularly noteworthy. We examined the distribution of the BamA peptides identified by MS and were struck by the heavy skewing towards the C-terminal β-barrel and the POTRA domain (POTRA5) in closest proximity to the barrel (Suppl. Figure 1)^49^. Sixteen of the 49 proteins identified in the Co-IP_Det were found in the Band_Det, including PrcA, four OBPs, NadN and BamA. As with the Co-IP_Det, the peptides recovered for PrcA in the Band_Det spanned the entire polypeptide (Suppl. Figure 1). The BamA peptides in the Band_Det showed the same skewing observed with the Co-IP_Det (Suppl. Figure 1).

**Table 1:** MS/MS analysis of the OM-MOSP complex.

Protein ID^1^	TDE designation^1^	Protein Name^1^	SignalP^2^	LipoP^3^	% Coverage (Co-IP_Det)^4^	Expectation (Co-IP_Det)^4^	% Coverage (Band_Det)^4^	Expectation (Band_Det)^4^
NP_971019.1	TDE0405	major outer sheath protein	Y	SpI	29.8	0.00E+00	93.2	0.00E+00
NP_973199.1	TDE2601	surface antigen (BamA)	Y	SpI	10	1.80E-29	21.9	6.50E-47
NP_973271.1	TDE2673	hypothetical protein	Y	SpI	12.3	3.10E-21	11.5	2.20E-14
NP_971372.1	TDE0761	protease complex-associated polypeptide (PrcA)	Y	SpII	13.6	7.50E-114	25.4	1.50E-62
NP_971880.1	TDE1273	oligopeptide/dipeptide ABC transporter peptide-binding protein (OppA/DppA)	Y	SpII	28.1	3.50E-104	27.9	1.10E-47
NP_972658.1	TDE2056	outer membrane hemin-binding protein A	Y	SpII	25.3	3.10E-103	35.2	7.70E-37
NP_972657.1	TDE2055	hemin-binding protein B	Y	SpII	27.5	1.10E-86	16.4	1.20E-16
NP_971680.1	TDE1072	lipoprotein (putative OppA/DppA)	Y	SpII	19.1	5.50E-66	49.3	0.00E+00
NP_972857.1	TDE2257	5′-nucleotidase	Y	SpII	14.3	2.90E-37	9.8	1.70E-11
NP_970756.1	TDE0139	hypothetical protein	Y	SpII	10.5	7.10E-25	11.2	8.70E-41
NP_971679.1	TDE1071	peptide ABC transporter peptide-binding protein (OppA)	Y	SpII	9.1	4.00E-18	71.4	0.00E+00
NP_973333.1	TDE2735	Leucine-rich repeat surface antigen	Y	SpII	5.4	5.30E-14	23.3	6.40E-41
NP_971012.1	TDE0398	oligopeptide/dipeptide ABC transporter periplasmic peptide-binding protein (DdpA/OppA)	Y	SpII	6.1	3.10E-10	17.5	8.70E-29
NP_972900.1	TDE2300	trypsin domain/PDZ protein, putative periplasmic serine protease	N	SpII	15.6	0.00E+00	26	1.60E-83
NP_971594.1	TDE0985	oligopeptide/dipeptide ABC transporter periplasmic peptide-binding protein (DdpA/OppA)	N	SpII	14.7	7.30E-46	70.1	0.00E+00
NP_972553.1	TDE1950	membrane lipoprotein TmpC	N	SpII	19.9	2.30E-32	66.1	1.60E-84
NP_973164.1	TDE2566	hypothetical protein	Y	SpI	13.2	1.00E-52		
NP_972968.1	TDE2369	hypothetical protein	Y	SpI	20.3	1.20E-40		
NP_971000.1	TDE0386	ABC transporter periplasmic substrate-binding protein	Y	SpI	29.9	2.70E-36		
NP_973106.1	TDE2508	hypothetical protein	Y	SpI	20.9	7.70E-28		
NP_973335.1	TDE2737	hypothetical protein	Y	SpI	22.4	4.90E-26		
NP_970939.1	TDE0325	hypothetical protein	Y	SpI	22.5	2.20E-21		
NP_971365.1	TDE0754	hypothetical protein	Y	SpI	14.4	2.00E-20		
NP_972321.1	TDE1717	hypothetical protein	Y	SpI	8.8	9.50E-17		
NP_972587.1	TDE1984	hypothetical protein	Y	SpI	11.4	2.20E-09		
NP_972452.1	TDE1848	hypothetical protein	Y	SpI	6.8	8.60E-08		
NP_972717.1	TDE2116	hypothetical protein	Y	SpI	4.1	3.30E-07		
NP_970802.1	TDE0186	hypothetical protein	Y	SpII	16.1	1.50E-43		
NP_972748.1	TDE2147	lipoprotein	Y	SpII	21.6	1.70E-40		
NP_971364.1	TDE0753	hypothetical protein	Y	SpII	18.4	2.20E-34		
NP_971343.1	TDE0731	hypothetical protein	Y	SpII	19.3	7.70E-33		
NP_971798.1	TDE1191	hypothetical protein	Y	SpII	17.7	1.20E-07		
NP_973205.1	TDE2607	hypothetical protein	Y	SpII	8.6	2.30E-07		
NP_973260.1	TDE2662	lipoprotein	Y	SpII	9.4	5.10E-07		
NP_973209.1	TDE2611	hypothetical protein	N	SpI	5.3	6.00E-14		
NP_972995.1	TDE2396	IM protein translocase component YidC	N	SpI	8.1	3.10E-12		
NP_971933.1	TDE1327	hypothetical protein	N	SpI	17.6	6.90E-12		
NP_972454.1	TDE1850	ABC transporter permease	N	SpI	7.9	7.10E-11		
NP_972908.1	TDE2308	hypothetical protein	N	SpI	10	2.10E-07		
NP_972248.1	TDE1642	hypothetical protein	N	SpII	36.1	1.60E-89		
NP_971413.1	TDE0803	hypothetical protein	N	SpII	25	1.60E-78		
NP_972817.1	TDE2217	galactose/glucose-binding lipoprotein (MglB)	N	SpII	20.1	3.80E-50		
NP_971044.1	TDE0430	TPR domain protein	N	SpII	18.9	3.70E-19		
NP_971629.1	TDE1021	lipoprotein	N	SpII	12.3	5.10E-18		
NP_971797.1	TDE1190	hypothetical protein	N	SpII	22.6	1.10E-12		
NP_972789.1	TDE2188	hypothetical protein	N	SpII	6	9.20E-07		
NP_970632.1	TDE0015	lipoprotein	N	SpII			28.1	9.20E-41
NP_971962.1	TDE1356	lipoprotein	N	SpII			16.6	3.80E-09
NP_972190.1	TDE1584	lipoprotein	N	SpII			41.8	3.10E-104
NP_972705.1	TDE2104	hypothetical protein	N	SpII			6.9	8.90E-13

^1^Protein IDs and annotations are based on the *T. denticola* ATCC 35405 RefSeq genome sequence^5^.

^2^Y is used to designate proteins identified by SignalP as containing an N-terminal cleaved signal sequence.

^3^SpI and SpII designate proteins identified by LipoP as containing N-terminal signal peptides with SPaseI and SpaseII cleavage sites, respectively.

^4^Co-IP_Aq refers to the eluate obtained from co-immunoprecipitation of the TX-114 aqueous phase using anti-MOSP^Fl^ antiserum. Band_Aq refers to the periplasmic-MOSP band (~400 kDa) excised from BN-PAGE gel.

MS of the Co-IP_Aq identified 20 candidate partners for periplasmic-MOSP, seven with annotations, and only three predicted lipoproteins (Table 2). Notably, four have putative functions related to protein folding, chaperoning, and/or quality control within the periplasm^50^. TDE1966 and TDE2300 are HtrA-like serine proteases, while TDE2602 is Skp. TDE1658, a member of the peptidylproyl isomerase (PPIase) superfamily, is annotated as “basic membrane protein (Bmp)” because of its relatedness to Bmp from *T. pallidum* protein^51^. Based on CDD search, TDE1658 contains a conserved domain found in PrsA, a factor required for folding and maturation of secreted proteins in Gram-positives^52^, but is atypical because it is not a lipoprotein. TDE1658 also contains an N-terminal domain found in SurA periplasmic chaperones^53^ (Suppl. Figure 2). Interestingly, phylogenetic analysis reveals that TDE1658 (and *T. pallidum* Bmp) lies somewhere between SurA and PrsA subfamilies (Suppl. Figure 3). The Co-IP-Aq also contained lone peptides for PrcA1 and PrcA2 (Suppl. Figure 1), almost certainly contaminants. MS of the excised periplasmic-MOSP band (Band_Aq) identified six candidate partners (Table 2). Of these, only TDE1658 was in the CoIP_Aq dataset; as with the Co-IP_Aq, the peptides were distributed over the entire polypeptide (Suppl. Figure 1). Lastly, BamA was identified in the Band_Aq. However, the distribution of BamA peptides in the Band_Aq differed markedly from those in the Co-IP_Det and Band_Det. All but two were from the POTRA arm, with a substantial number derived from POTRA1 and POTRA2, the domains most distal from the C-terminal, OM-embedded β-barrel (Suppl. Figure 1).

**Table 2:** MS/MS analysis of the periplasmic MOSP complex.

Protein ID^1^	TDE designation^1^	Protein Name^1^	SignalP^2^	LipoP^3^	% Coverage (Co-IP_Aq)^4^	Expectation (Co-IP_Aq)^4^	% Coverage (Band_Aq)^4^	Expectation (Band_Aq)^4^
NP_971019.1	TDE0405	Major outer sheath protein	Y	SpI	14.5	2.90E-35	88.4	0.00E+00
NP_972263.1	TDE1658	Basic membrane protein (SurA/PrsA)	Y	SpI	37.4	0.00E+00	48.6	8.70E-62
NP_970715.1	TDE0098	Hypothetical	Y	SpI	20.5	2.50E-80		
NP_972810.1	TDE2210	Hypothetical	Y	SpI	13.1	2.10E-51		
NP_971082.1	TDE0468	Hypothetical	Y	SpI	40.1	5.00E-51		
NP_972660.1	TDE2058	Hypothetical	Y	SpI	24.3	2.00E-38		
NP_972968.1	TDE2369	Hypothetical	Y	SpI	27.1	2.20E-37		
NP_971844.1	TDE1237	Hypothetical	Y	SpI	5.7	2.70E-12		
NP_972461.1	TDE1857	Hypothetical	Y	SpI	22.5	8.70E-11		
NP_971372.1	TDE0761	Protease complex-associated polypeptide (PrcA)	Y	SpII	8.6	2.40E-31		
NP_972949.1	TDE2350	Lipoprotein	Y	SpII	17.8	7.60E-19		
NP_971139.1	TDE0525	Hypothetical	N	SpI	28.8	3.00E-103		
NP_972332.1	TDE1728	Hypothetical	N	SpI	22.8	3.30E-77		
NP_973297.1	TDE2699	Hypothetical	N	SpI	22.2	5.40E-65		
NP_971277.1	TDE0664	OmpA family protein	N	SpI	18.5	7.40E-62		
NP_971903.1	TDE1297	LysM/M23/M37 peptidase	N	SpI	17.3	3.30E-38		
NP_972569.1	TDE1966	Trypsin domain/PDZ (HtrA1)	N	SpI	10.6	2.50E-28		
NP_973200.1	TDE2602	Putative outer membrane chaperone (Skp)	N	SpI	35.4	5.10E-25		
NP_971555.1	TDE0945	Hypothetical protein	N	SpI	8.1	3.10E-16		
NP_972453.1	TDE1849	Hypothetical protein	N	SpI	10.2	2.40E-08		
NP_972900.1	TDE2300	Trypsin domain/PDZ protein (putative periplasmic HtrA-like serine protease)	N	SpII	12.4	3.50E-35		
NP_973199.1	TDE2601	Surface antigen (BamA)	Y	SpI			13.1	7.00E-29
NP_971889.1	TDE1282	Hypothetical	Y	SpI			13.8	3.50E-07
NP_971359.1	TDE0748	ABC transporter periplasmic iron compound-binding protein	Y	SpI			12.5	9.40E-07
NP_972765.1	TDE2164	Hypothetical protein	Y	SpII			43.4	4.40E-13

^1^Protein IDs and annotations are based on the *T. denticola* ATCC 35405 RefSeq genome sequence^5^.

^2^Y is used to designate proteins identified by SignalP as containing an N-terminal cleaved signal sequence.

^3^SpI and SpII designate proteins identified by LipoP as containing N-terminal signal peptides with SPaseI and SpaseII cleavage sites, respectively.

^4^Co-IP_Aq refers to the eluate obtained from co-immunoprecipitation of the TX-114 aqueous phase using anti-MOSP^Fl^ antiserum. Band_Aq refers to the periplasmic-MOSP band (~400 kDa) excised from BN-PAGE gel.

### Dentilisin components interact predominantly with the larger OM-MOSP complex

**Figure 8:**
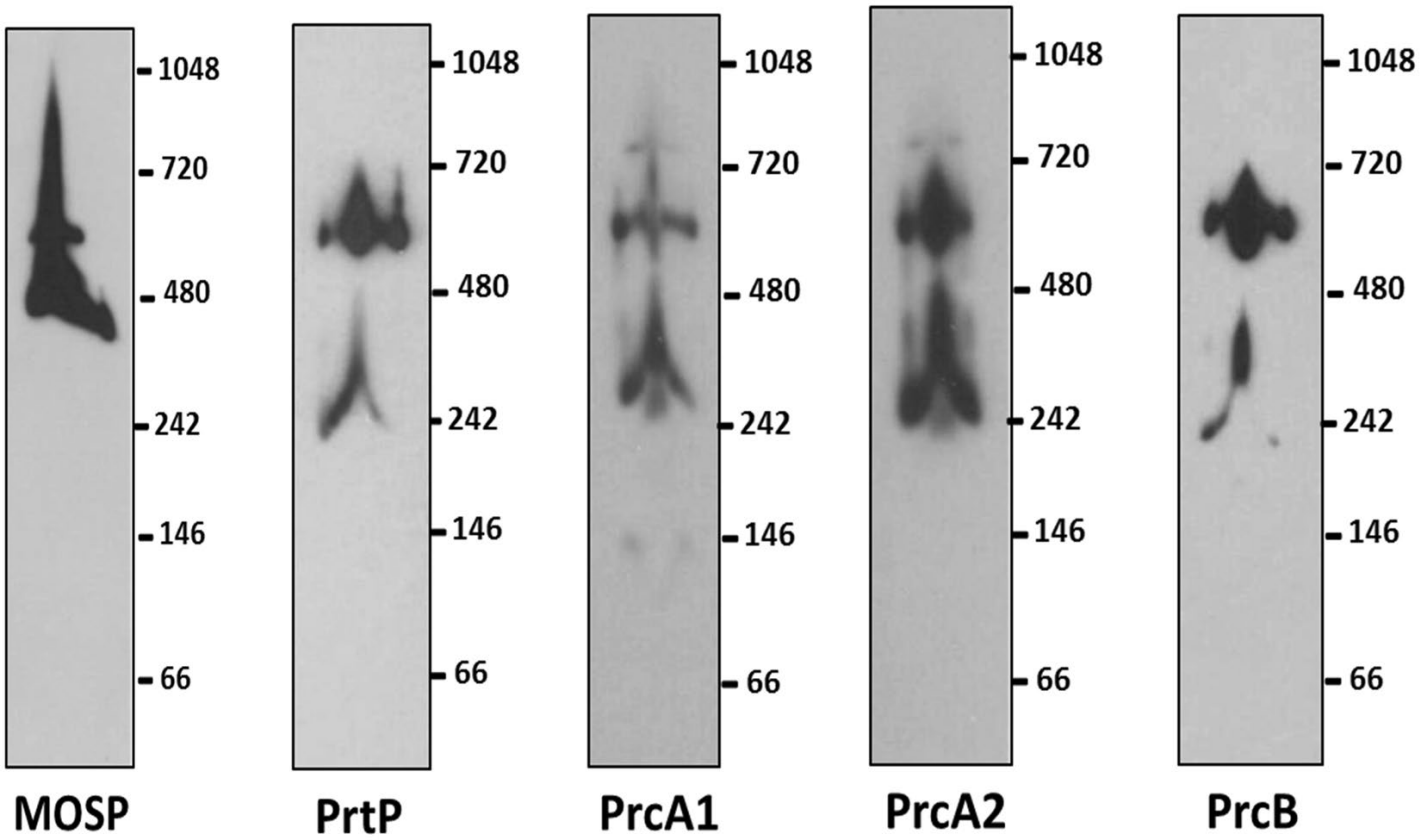
Dentilisin associates predominantly with the larger OM-MOSP complex. BN-PAGE of the NP-40 supernatant containing the OM-MOSP immunoblotted with rat antiserum directed against MOSP^Fl^ and rabbit antisera against PrtP, PrcA1, PrcA2 and PrcB. Molecular mass standards (kDa) are indicated on the right of each gel.

We immunoblotted a BN-PAGE gel of the NP-40 supernatant with antisera against different dentilisin components, as well as MOSP, to ascertain which of the two OM-MOSP complexes interacts with the protease. A diffuse band migrating below the ~480 kDa MOSP complex, centered at ~240 kDa, reacted with all four dentilisin antisera and is likely the unassociated dentilisin complex (Fig. 8). The four dentilisin antisera predominantly recognized the larger OM-MOSP complex.

## Discussion

OMPs fulfill myriad homeostatic functions in diderm bacteria, including passive and active import of nutrients, cell communication, efflux of toxic molecules, and adhesion, in addition to biogenesis of the OM. OMPs also play numerous roles as virulence determinants. This functional diversity reflects the versatility of the amphiphilic β-barrel structure^54^. Recently, investigators have turned their attention to understanding how concepts of OMP structure, function and localization developed with Gram-negatives apply to phylogenetically and morphologically divergent diderms, such as *T. denticola*, an oral commensal strongly associated with periodontal disease^8^.

We performed modeling of MOSP^Fl^ using I-TASSER to extend bioinformatics predictions that MOSP contains distinct MOSP^C^ and MOSP^N^ domains. The low C-score values likely reflect the unavailability of protein structures homologous to MOSP. Further biophysical studies of individual recombinant MOSP domains validated these *in silico* analyses. The use of large and small nanodiscs^41,42^ was particularly informative by demonstrating that MOSP^C^ can stably trimerize and insert into lipid bilayers as either monomers or pre-formed trimers. Further structural and biophysical characterization will be needed to explain how the MOSP^C^ β-barrel, predicted to have 10 transmembrane strands, forms a channel with efflux properties comparable to the 16-stranded *E. coli* OmpF, its functional ortholog^10^. The MS data pointing to physical interactions between OM-MOSP and the BamA β-barrel constitute strong, albeit indirect, evidence that OM insertion of MOSP^C^ is BAM-dependent. Although the subunits of *T. denticola*’s BAM apparatus differ from those of *E. coli*, the mechanism, termed β-augmentation, by which the BamA β-barrel guides nascent OMPs into the OM bilayer, is probably highly similar in both organisms^55^. Thus, for MOSP^Fl^ to achieve its bipartite architecture, MOSP^C^ should contain the information needed to direct barrel closure *in vivo*, an assumption strongly supported by *in vitro* folding data.

In contrast to MOSP^C^, MOSP^N^ forms an extended, hydrophilic monomer analogous to TprF, a truncated MOSP ortholog located within the periplasm of *T. pallidum* ^25^. In concert with IFA results showing that only MOSP^C^ is surface-exposed, our collective findings indicate a topology in which MOSP^N^ projects into the periplasm. It should be noted that an identical topology has been established for *T. pallidum* Tpr paralogs that contain both MOSP^N^ and MOSP^C^ domains^24,25^. According to this bipartite topologic model, MOSP^N^ would be available to serve as a periplasmic scaffold; using a variation of BN-PAGE, Fenno and co-workers^23^ proposed that MOSP may bind proteins in addition to dentilisin. Partnering of OM-MOSP with SBPs of ABC transporters, strongly suggested by the MS data, would physically link the OM channels that import exogenous nutrients with the permeases that transport them into the cytosol. That multiple oligopeptide-binding proteins are among the candidate OM-MOSP partners seems significant given the ability of *T. denticola* to ferment amino acids as a sole energy source^5^.

A central tenet of OM biogenesis is that newly exported OMPs exist in the periplasm as intermediates maintained in a protected, folding-competent state by the chaperones, primarily SurA, but also Skp and DegP, that escort them to the BAM complex^37^. Evidence presented herein reveals that MOSP only partially adheres to this paradigm, a situation, to the best of our knowledge, unprecedented with Gram-negatives. Indeed, we found a MOSP^Fl^ conformer that forms highly-stable core trimers within the periplasm. Unlike intact organisms, which labeled with antibodies to MOSP^C^, but not MOSP^N^, in IFA experiments, treponemes whose membranes had been disrupted by detergent labeled with antibodies to both domains, consistent with the presence of a full-length periplasmic form, as previously reported^10^. Under conditions in which surface-exposed MOSP^C^ was PK-susceptible (leaving a residual band reactive with anti-MOSP^N^ but not anti-MOSP^C^), a full-length form of the protein remained inaccessible to the enzyme. Importantly, TX-114 phase partitioning demonstrated the co-existence of MOSP^Fl^ proteins with markedly different physical properties. DDM also solubilized trimers of both conformers that could be separated by subsequent phase partitioning. Lastly, it was possible to selectively solubilize the OM-MOSP conformer, leaving periplasmic MOSP bound to the protoplasmic cylinder but extractable into the TX-114 aqueous phase.

What, then, is the periplasmic conformer and how is it generated? One obvious possibility, suggested by the presence of Skp and the POTRA arm of BamA in the aqueous Co-IP MS dataset, is that it consists of aggregated or misfolded OM-MOSP that either failed to access or “fell off” the BAM pathway. However, misfolded OMPs typically are targeted for degradation by periplasmic “watch dogs”, such as HtrA proteases^50,56^, which we also found in the aqueous Co-IP MS dataset. The large amounts of soluble, intact periplasmic-MOSP in *T. denticola* in the TX-114 aqueous phase strongly argues against misfolding and aggregation. Two lines of evidence lead us to postulate that periplasmic-MOSP arises from a previously unrecognized folding pathway not present in prototypical Gram-negatives. One is the finding that MOSP^Fl^ expressed with a PelB signal sequence localizes exclusively to the OM in *E. coli*. In addition to supporting the treponeme specificity of the pathway that gives rise to periplasmic-MOSP, these results clearly demonstrate conservation of the protein-encoded signals required to engage the BAM pathway despite the phylogenetic divide separating *E. coli* and *T. denticola*. The other is the discovery that the periplasmic-MOSP complex contains TDE1658, a member of the PPIase superfamily that includes SurA chaperones (‘holdases’) and PrsA foldases^52,53,57^. As a SurA, one would expect TDE1658 to bind unfolded MOSP^C^, protecting it from misfolding and degradation; however, one also would expect it to shepherd MOSP to the BAM pathway, which clearly does not occur with the periplasmic conformer. As a foldase, TDE1658 would direct MOSP^C^ into an alternative conformation that can still trimerize and attract a distinct set of interaction partners. SurA chaperones and PrsA foldases interact transiently with their partners^52,53,57^. Thus, another novel feature of TDE1658’s proposed activity as a SurA/PrsA functional chimera is that it seems to associate permanently with its target, at least with respect to MOSP. Whether TDE1658 fulfills a conventional SurA function in OM biogenesis in *T. denticola* remains to be determined. Lastly, we can only speculate as to the biological purpose(s) of periplasmic-MOSP and the alternative pathway. One possibility is cell envelope housekeeping, serving as a platform for removal of unwanted proteins or protein degradation products, a function that would explain the ostensible association of periplasmic-MOSP with HtrA proteases. Another, which is not mutually exclusive, is as a gatekeeper regulating the entrance of folding-competent MOSP into the BAM pathway. Additional experimentation will be required to definitively identify the interacting partners with both MOSP conformers and further clarify the function of periplasmic MOSP.

The dentilisin protease complex is considered a key player in the progression of periodontal disease^6,7,29,30^. Not as well recognized is its probable role in providing peptides to meet the spirochete’s biosynthetic and energy requirements. For years, it was believed that dentilisin consists of the lipoprotein PrtP and the PrtP-mediated cleavage products of another lipoprotein, PrcA (*i.e*., PrcA1 and PrcA2)^31–34^. However, Godovikova *et al*.^35^ reported a third lipoprotein constituent, PrcB, a finding we confirmed by immunoblot and co-IP. By SDS-PAGE, the complex migrates at ~100 kDa, considerably below the combined masses of the individual components (~150 kDa), suggesting a compact, hetero-oligomeric structure. Prior to assembly, PrtP is cleaved at residue 169, releasing an ~16 kDa acylated proteolytic product (designated PrtN)^23,32^. Thus, only two of the four components, PrcA1 and PrcB, provide N-terminal lipid anchors for the mature complex. Results here and elsewhere^23,31,32^ show that dentilsin is resistant to dissociation in high concentrations of SDS unless boiled. Because the polypeptide moieties of lipoproteins are almost always hydrophilic, PrtP and PrcA2 likely partition into the TX-114 detergent phase because they are bound to PrcB and PrcA1 following cleavage.

We also confirmed that the mature dentilisin complex associates with MOSP^11,12,23^. In doing so, we made the novel, but not unexpected, observation that this interaction exclusively involves the OM conformer. Previously, it was reported that MOSP could be pulled down with PrcA2 antiserum but not with antisera to other components of dentilisin^23^. This finding is reminiscent of our own MS results identifying only peptides from PrcA1 and PrcA2 in association with OM-MOSP. We surmise that PrcA1 and PrcA2 are OM-MOSP’s primary partners and that SDS dissociates the relatively weak interactions between the two, yielding MOSP trimers and dentilisin hetero-oligomers. Presumably, the association of MOSP and dentilisin follows the formation and OM localization of the individual complexes, a process that somehow prevents individual dentilisin components from interacting with periplasmic-MOSP following export across the cytoplasmic membrane. Importantly, BN-PAGE revealed that the interactions between OM-MOSP and dentilisin and the assembly of MOSP-dentilisin complexes are more complicated than the convergence of pathways implied by the ability of MOSP antiserum to pull down dentilisin. Similar to Godovikova and co-workers^23^, we found a freestanding subpopulation of dentilisin with the remainder bound to the larger, lower abundance MOSP complex. These results add another level of complexity requiring further investigation to a protein already well recognized for its multi-functionality in the pathogenesis of periodontal disease.

## Materials and Methods

### Ethics Statement

Animal protocols described in this work strictly follow the recommendations of the Guide for Care and Use of Laboratory Animals from the National Institutes of Health and were approved by the University of Connecticut Health Center Animal Care Committee under the auspices of Public Health Service (PHS) Animal Welfare Assurance A3471-01.

### Propagation of *T. denticola*

*T. denticola* (ATCC 35405) was grown from mid- to late-logarithmic phase in new oral spirochete broth (NOS) supplemented with 10% heat inactivated normal rabbit serum at 37 °C in a closed chamber with a GasPak (Becton Dickinson, USA) as previously described^26^.

### Cloning of DNAs encoding MOSP, MOSP^N^, MOSP^C^, *T. denticola* Skp and *T. pallidum* TprF (TP0316)

Cloning of DNAs encoding MOSP^Fl^ without its signal sequence, MOSP^N^, MOSP^C^, and *T. pallidum* (Nichols) TprF without its signal sequence^25^ into pET23b was described previously^10,25^. To promote incorporation of MOSP^Fl^ into the outer membrane of *E. coli*, the DNA encoding MOSP^Fl^ without its signal sequence was cloned into the BamHI and HindIII restriction sites of pET20b (Novagen, USA) and expressed with an N-terminal PelB signal sequence. DNA encoding *T. denticola* Skp (TDE2602/NP_973200.1) without the signal sequence was PCR-amplified and cloned into the BamHI and HindIII restriction sites of pET23b (Novagen, USA).

### Expression, purification and refolding of recombinant proteins

All proteins were expressed in *E. coli* OverExpress™ C41 (DE3) (Lucigen/VWR, Radnor, PA) at 37 °C. Proteins were induced with 1 mM IPTG once cultures reached an OD_600_ of 0.5. Following induction, cells were harvested at 7,000 × g for 20 min at 4 °C. The pellet was lysed in Tris-NaCl buffer (20 mM Tris-HCl [pH 8.0], 150 mM NaCl) supplemented with protease inhibitor cocktail (PIC; Sigma-Aldrich). The lysate was centrifuged at 12,000 rpm for 40 min at 4 °C to separate insoluble proteins and cell debris. The supernatant was mixed with Ni-NTA resin (Qiagen, USA) for 1 h, washed with Tris-NaCl buffer followed by another wash with the same buffer containing 20 mM imidazole. Elution was carried out in Tris-NaCl buffer with 400 mM imidazole at pH 8. For purification of denatured protein, the insoluble pellet was dissolved in Tris-NaCl buffer containing 6 M guanidine-HCl and left on a shaker for 2 h at room temperature (RT). Any insoluble material was separated by centrifugation at 12,000 rpm for 40 min at 4 °C, and the supernatant was mixed with Ni-NTA resin for 2 h. The resin was washed in Tris-NaCl buffer containing 8 M urea at pH 8.0 and further washed with the same buffer containing 20 mM imidazole. Elution was carried out in Tris-urea buffer (20 mM Tris-HCl [pH 8.0], 8 M urea, 400 mM imidazole).

To refold denatured MOSP^C^, eluate containing purified MOSP^C^ was concentrated to 0.1 mM and added in a dropwise fashion at RT into a 20-fold excess of 1.5% (n-Dodecyl-β-D-Maltoside (DDM; Anatrace, USA) dissolved in 15 mM Tris buffer at pH 9.0 with 2 mM EDTA; the efficiency of refolding was assessed by CD spectroscopy^10^ (see below). To refold denatured MOSP^N^, eluate containing purified MOSP^N^ was concentrated to 0.05 mM and dialyzed at 4 °C in a stepwise manner against 8, 6, 4, 2, 1, 0.5 and 0 M urea in 20 mM Tris-HCl, 250 mM NaCl, 5% glycerol, 2 mM EDTA with PIC, pH 8.5. The refolded protein was purified by SEC on a S200 column (GE, USA) equilibrated with Tris-NaCl buffer, pH 7.5.

### Far-UV circular dichroism spectroscopy

Far-UV circular dichroism (CD) spectroscopy was performed using a JASCO J-715 spectral polarimeter as described previously^10,24,25^.

### Preparation of nanodiscs and examination by transmission electron microscopy

Large and small nanodiscs were prepared using MSP1E3D1^58^ and a truncated construct (D7) derived from MSP1D1^44^. DNAs encoding both constructs were cloned into pET28a for expression in *E. coli* BL21 (DE3) and purified using the protocol developed by Sligar’s group^41^. Nanodiscs were formed in the presence of 1,2-dimyristoyl-sn-glycero-3-phosphocholine (DMPC; Avanti Polar, USA) lipids at RT. D7 or E3D1 were mixed with refolded MOSP^C^ at a D7/E3D1-to-MOSP^C^ ratio of 2:1. DMPC lipids were then added in a molar excess of 20 and 100 for D7 and E3D1, respectively. The reaction mixture was kept on a shaker for 1 h, after which SM2 Bio-Beads (Bio-Rad, USA) were added to the mixture and left gently suspended overnight. Beads were filter-separated from the reaction mixture and further subjected to SEC on S200 column (GE, USA) equilibrated with Tris-NaCl buffer at pH 7.5. Peak fractions containing MOSP^C^ incorporated into the discs were used for transmission electron microscopy (TEM), performed at the Biosciences Electron Microscopy Facility of the University of Connecticut. Discs containing MOSP^C^ were diluted several-fold in water to obtain a well-dispersed population, applied to a glow discharged, carbon-coated, 400-mesh copper grid (Ted Pella Inc., USA), and negatively stained with freshly prepared 0.75% uranyl formate (SPI-Chem, USA). TEM images were taken on a Tecnai G2 Spirit BioTWIN microscope (FEI, USA) at an accelerating voltage of 80 kV with a defocus of ∼−1.6. Refolding efficiency was assessed through far-UV CD spectroscopy; images of the nanodiscs were obtained by transmission electron microscopy (TEM) and averaged over 70–80 discs/image using the Xmipp software suite^59^.

### Immunologic reagents and immunoblot analysis

Rat polyclonal antisera directed against *T. denticola* Skp was generated using purified, recombinant His-tagged Skp (see above). Rat polyclonal antisera directed against MOSP^Fl^, MOSP^N^ and MOSP^C^, *E. coli* OmpA, *E. coli* Skp, α-ATPB (ATP synthase subunit A; Abcam), and isolated *T. denticola* periplasmic flagella were described previously^10,25,26^. Rabbit antibodies against PrtP, PrcA1, PrcA2, PrcB and MOSP^Fl^ ^23,33,35^ were a generous gift from Dr. Christopher Fenno (University of Michigan, Ann Arbor). Immunoblot analysis was performed on samples separated by 12% SDS or 4–16% BN-PAGE. Proteins were transferred from the gel to nitrocellulose membrane (0.45 µM pore size, GE) using a semi-dry (Bio-Rad) or wet apparatus (XCell surelock blot module, Invitrogen). Membranes were blocked for 1 h in PBS with 5% nonfat dry milk, 3% BSA, and 0.1% Tween 20 and probed overnight at 4 °C with primary antibodies directed against specific proteins. Rat primary antibodies directed against Skp, MOSP^Fl^, MOSP^C^, and MOSP^N^ were used at a dilution of 1:5,000; rabbit antibodies directed against PrtP, PrcA1, PrcA2 and PrcB were used at a dilution of 1:10,000. After washing with PBS with 0.1% Tween 20 (PBST), the membranes were incubated for 1 h at RT with horseradish peroxidase (HRP)-conjugated goat anti-rat (Southern Biotech, USA) or anti-rabbit (Bio-Rad, USA) antibody at a dilution of 1:30,000. Following washes with PBST, the blots were developed using the SuperSignal West Pico chemiluminescent substrate (Thermo Fisher Scientific).

### Analytical Ultracentrifugation

Sedimentation velocity experiments were performed using a Beckman-Coulter XL-I analytical ultracentrifuge at 20 °C and 40,000 rpm. Samples containing approximately 0.05 mM purified, refolded MOSP^N^ and TprF in Tris-NaCl buffer pH 7.5 were loaded into double sector cells equipped with 1.2 cm Spin60 charcoal-epon centerpieces (Spin Analytical, Berwick, ME) and quartz windows. The raw scans were recorded using absorbance optics at 280 nm. The buffer densities and viscosities were calculated using SEDNTERP^60^. Initially, the sedimentation velocity data was analyzed using the time derivative method^61^ in DCDT+^62^ to ensure that the samples contained homogeneous single species. To obtain the sedimentation coefficients and the buoyant molar masses of the proteins, the data was fit to a non-interacting discrete single species model using a c(s) distribution model with SEDFIT^63^.

### Triton X-114 phase partitioning

Triton X-114 (TX-114) (Sigma-Aldrich, USA) phase partitioning was performed as described previously^10,24,46^. 10 µg of recombinant protein or 2 × 10^9^ *T. denticola*, cultivated as described above, were added to 2% TX-114 in PBS supplemented with 0.5% PIC and incubated overnight at 4 °C. The insoluble fraction was separated by centrifugation at 18,000 × g for 20 min. at 4 °C, following which the supernatant was further subjected to phase separation. The resulting detergent-enriched and aqueous phases subsequently were extracted four more times with PBS or 2% TX-114, respectively. All samples were precipitated overnight with 10 volumes of acetone at −20 °C for subsequent analysis by SDS-PAGE and immunoblotting.

To obtain DDM-solubilized MOSP for TX-114 phase partitioning, 2 × 10^9^ *T. denticola* were resuspended in Tris-NaCl buffer and disrupted by sonication on ice for three 20 sec pulses interspersed with 30 sec intervals. The lysate was centrifuged at 1,000 × g for 10 min at 4 °C to remove cell debris and further fractionated by ultracentrifugation at 100,000 × g for 45 min at 4 °C. The pellet containing the membrane fraction was solubilized in 2% DDM and then subjected to TX-114 phase partitioning as explained above (also see Fig. 4B).

### Proteinase K accessibility experiments with *T. denticola*

2 × 10^9^ *T. denticola* were harvested and reconstituted in 50 mM Tris-HCl (pH 8) and 10 mM CaCl_2_. Proteinase K (Promega) was added at a working concentration of 100 µg/ml and left at 40 °C for 2 h. The reaction was quenched with 5 mM PMSF and separated on SDS-PAGE after quickly heating the sample for 10 min. Subsequent immunoblot analyses were carried out using rat polyclonal antibodies directed against MOSP^Fl^, MOSP^C^ or MOSP^N^.

### Separation of native OM and periplasmic MOSP conformer complexes

Freshly harvested *T. denticola* (2 × 10^9^ cells/ml) were dissolved in Pierce® IP Lysis Buffer (25 mM Tris-HCl [pH 7.4] 140 mM NaCl, 1% Nonidet P-40 (NP-40), 1 mM EDTA, 5% glycerol) (Thermo Scientific Pierce, USA) and left on a shaker for 2 h at 4 °C. The detergent-solubilized material was subjected to ultracentrifugation at 100,000 × g for 45 min at 4 °C. The supernatant was clarified on a Superdex S200 (10/300) column (GE, USA) where the MOSP-containing fraction was obtained in the void volume. The water-soluble (aqueous/periplasmic) conformer of MOSP was obtained by TX-114 phase partitioning; several aqueous phases were pooled, concentrated and clarified through S200 column obtaining the higher molecular weight MOSP in the void volume (Fig. 6C).

### Assessment of MOSP trimerization

Duplicate samples of the NP-40 solubilized periplasmic and OM conformers obtained by TX-114 phase partitioning or DDM-solubilized MOSP (above), respectively, were mixed with 1X Laemmli sample buffer (Bio-Rad, USA). One set was boiled for 10 min while the other was left at RT. Both sample sets were subsequently separated by SDS-PAGE (12.5% polyacrylamide gels) and immunoblotted with rat anti-MOSP^Fl^.

### Blue Native-polyacrylamide gel electrophoresis (BN-PAGE)

Samples for BN-PAGE were prepared as described previously^24^ and resolved in a 4–16% Bis-Tris acrylamide gel (Invitrogen) at 4 °C using the BN-PAGE method of Wittig *et al*.^64^. The cathode buffer (50 mM Tricine, 15 mM Bis-Tris, pH 7.0) contained 0.02% Coomassie Brilliant Blue G-250 (CBB-G250) for the first 1/3^rd^ of the run, after which the gel was run with fresh cathode buffer without CBB-G250. For the duration of the run, the anode buffer consisted of 50 mM Bis-Tris (pH 7.0). Resolved lysates were transferred to a nitrocellulose membrane in 50 mM Tricine (pH 7.0) at 25 V for 2–3 h followed by immunoblotting with respective antibodies.

### Co-immunoprecipitation (Co-IP) assays

500 µl of NP-40 supernatant and TX-114 aqueous phase containing the OM and periplasmic conformers, respectively, were mixed with rat anti-MOSP^Fl^ antisera and 10 µl of PIC and left rocking overnight at 4 °C. The following day, Protein G magnetic beads (EMD-Millipore, USA) were added; after 1 h of incubation with rocking at 4 °C, material bound to the beads was eluted with 0.2 M glycine-HCl (pH 2.2). Similar steps were performed with anti-MOSP^Fl^ antisera crosslinked to agarose resin obtained using the Crosslink IP kit (Pierce, USA). Eluted samples were neutralized by 1 M Tris-HCl pH 8.5 and analyzed by SDS-PAGE and immunoblotted with rabbit polyclonal antibodies against PrtP, PrcA1, PrcA2, PrcB and MOSP^Fl^ followed by detection with HRP conjugated Clean-Blot IP detection reagent (Thermo Scientific, USA). Additionally, the NP40-solubilized MOSP (detergent conformer) was mixed with 2% DDM for 2 h at RT and co-immunopreciptated using MOSP^Fl^ antibodies using Protein G magnetic beads. The eluate was probed with rabbit antisera against MOSP^Fl^ and PrtP using the Clean-Blot IP detection reagent (Thermo Scientific, USA).

### Gel microdroplet immunofluorescence assays with *T denticola*

Mid-late logarithmic-phase *T. denticola* were encapsulated in low-melting-point agarose microdroplets^26,45^ and washed extensively with DMEM (Thermo-Fisher, USA) prior to the addition of primary antibodies. Beads then were resuspended in DMEM with 2% BSA and 1:200 dilutions of rat antisera against MOSP^N^, MOSP^C^, or *T. denticola* periplasmic flagellar filaments in the presence or absence of 0.05% (v/v) Triton X-100^26^. After overnight incubation with gentle mixing at 4 °C, beads were washed three times by low-speed centrifugation (500 × g) and incubated for 2 h at RT with DMEM containing 2% BSA and 1 µg/ml of goat anti-rat Alexa Fluor 488. Following further washing with DMEM, the gel microdroplets were mounted onto glass slides with Vectashield® anti-fade reagent (Invitrogen) containing DAPI. Fluorescent images were acquired on an epifluorescence Olympus BX-41 microscope using a 100X (1.4 NA) oil immersion objective equipped with Retiga Exicharge-coupled-device (CCD) camera (Q Imaging, USA) and DAPI and fluorescein isothiocyanate (FITC) filter set. The data were analyzed using Cell M (Olympus) and ImageJ.

### Fractionation of *E. coli* expressing MOSP^Fl^ with a PelB signal sequence

DNA encoding the *msp* gene without its native signal sequence was cloned into the BamHI and HindIII sites of pET20b vector (EMD Millipore) downstream and in-frame with an N-terminal PelB signal sequence^25^. Recombinant PelB-MOSP was expressed in *E. coli* OverExpress™ C41 (DE3) at 20 °C without induction in LB medium supplemented with 50 μg/ml ampicillin. Fractionation of *E. coli* was based on a previously published protocol^65^. Briefly, 1 L of cells were pelleted and resuspended in 10 mL of 200 mM Tris-HCl (pH 8.0), 1 M sucrose, 1 mM EDTA; lysozyme was added to a final concentration of 1 mg/ml^65^. The suspension was mixed and incubated for 10 min at RT followed by the addition of 40 mL of ultrapure deionized water (dH2O) and placed on ice; the cells were then centrifuged at 200,000 × g for 45 min at 4 °C. The supernatant, which contained the periplasmic fraction was removed. The pellet, consisting of spheroplasts, was resuspended in 5 mL ice-cold 20 mM Tris-HCl (pH 7.5), 5 mM EDTA, 0.2 mM DTT supplemented with 50 μL DNase (1 mg/mL). The spheroplasts were ruptured in a French Press with three passes at 108 Pa. Unbroken cells were removed by centrifugation at 5000 × g for 10 min at 4 °C. The supernatant, containing cytosolic and crude membrane fractions, was centrifuged at 300,000 × g for 2 h at 4 °C. The supernatant (cytosolic fraction) was removed, and the pellet (crude membrane) was resuspended in 10 mL of 50 mM Tris-HCl (pH 7.5), 2% (w/v) Triton X-100, 10 mM MgCl_2_ and centrifuged at 90,000 × g for 30 min at 4 °C. The supernatant (inner membrane) was removed. The pellet (outer membrane) was washed in 2 mL of 50 mM Tris-HCl (pH 7.5), 2% (w/v) Triton X-100, 10 mM MgCl_2_; centrifuged at 90,000 × g for 30 min at 4 °C, washed three times in 1 mL of dH2O, and frozen at −20 °C prior to SDS-PAGE and immunoblot analysis. To assess the multimeric state of OM-localized PelB-MOSP, the OM fractions were extracted in 2% DDM; the supernatant obtained following ultracentrifugation was examined by BN-PAGE and immunoblotting.

### Immunofluoresence analysis of *E. coli* expressing exported MOSP^Fl^

*E. coli* expressing PelB-MOSP (see above) were harvested and washed repeatedly with PBS and fixed in 4% paraformaldehyde for 30 min at RT. Permeabilization was achieved by incubating the cells with Buffer P (PBS with 0.1% Triton-X100, 10 mM EDTA) containing 100 μg/ml lysozyme for 30 min at RT. Both intact and permeabilized samples were resuspended in PBS or Buffer P respectively containing 2% BSA prior to incubation with 1:1,000 dilutions of rat anti-MOSP^C^, MOSP^N^ or Skp (*E. coli*) for 2 h at 4 °C, followed by incubation with a 1:300 dilution of goat anti-Rat AlexFluor 488 for 1 h. Following washing, cells were mounted onto glass slides with Vectashield® containing DAPI. Fluorescent images were acquired on an epifluorescence Olympus BX- 41 microscope using a 40X objective equipped with Retiga Exicharge-coupled-device (CCD) camera (Q Imaging, USA) and DAPI and fluorescein isothiocyanate (FITC) filter sets. The data were analyzed using Cell M (Olympus) and ImageJ.

### Mass spectrometric analysis

The NP-40 supernatant and TX-114 aqueous phase containing the OM and periplasmic conformers, respectively, were fractionated by BN-PAGE following which proteins were visualized using a mass spectrometry-compatible silver stain (PIERCE, USA). Bands corresponding to the ~480- and ~400-kDa MOSP complexes, determined by immunoblotting, were excised. In separate experiments, eluates obtained with 0.2 M glycine-HCl (pH 2.2) elution from pull-down experiments of NP-40 supernatant and TX-114 aqueous phase using agarose-crosslinked MOSP^Fl^ antiserum (described above) were neutralized with 1 M Tris-HCl (pH 8.5), precipitated with acetone, and reconstituted in buffer compatible with proteolysis. Both the excised bands and eluates were sent to the W.M. Keck Foundation Biotechnology Resource Laboratory at Yale University. LC MS/MS was performed on the proteolytically digested samples using LTQ-Orbitrap XL mass spectrometer (Thermo Scientific, USA) equipped with Waters nanoAQUITY ultra high-pressure liquid chromatographs (UPLC) for peptide separation. MS/MS spectra were searched using Mascot against a custom configured *T. denticola* database.

### Bioinformatics and Phylogeny

Non-redundant database searches were performed using BLASTP^66^. Conserved protein domains were identified using NCBI CDD-Search^67^. Proteins used for phylogenic analysis of TDE1658 were obtained from the UniProtKB database^68^ using the search terms “SurA” and “PrsA” and filtered to include only reviewed records. Closely-related sequences (>65% identity) were removed using CD-HIT^69^. The list of selected sequences was refined further using a guide tree generated in Clustal Omega^70^ to remove orthologues for the ribosome-associated chaperone Trigger Factor, which formed a separate cluster. Putative SurA/PrsA orthologs from *Treponema* sp. and *Borrelia burgdorferi* were obtained from UniProtKB. The *Leptospira interrogans* SurA ortholog was identified by Giuseppe *et al*.^71^. Multiple sequence alignment of the sequences was performed using MUSCLE with default parameters^72^. Phylogenetic analyses were carried out using the PHYLIP 3.696 package^73^. Pairwise sequence distance matrix was computed using Henikoff/Tillier Probability Matrix from Blocks (PMB) matrix in ProtDist and trees were constructed using Fitch with global rearrangements. Confidence levels for the bifurcating branches were obtained using Seqboot program with 1000 step bootstrapping. Phylogenetic trees were visualized by using iTOL^74^.

### Data availability

All data generated or analyzed during this study are included in this published article and its supplementary information files.

## Electronic supplementary material

Supplementary Figures 1-4

Supplementary Table 1

Supplementary Table 2

Supplementary Table 3

Supplementary Table 4

Supplementary Table 5

Supplementary Table 6

Supplementary Table 7

Supplementary Table 8
