# Supplementary material for: The major outer sheath protein forms distinct conformers and multimeric complexes in the outer membrane and periplasm of Treponema denticola
Source: Sci Rep. 2017 Oct 16;7:13260. doi: 10.1038/s41598-017-13550-6 (PMC5643300; doi:10.1038/s41598-017-13550-6)
Supplement: Supplementary file 1 — Supplementary Figures 1-4 [file 41598_2017_13550_MOESM1_ESM.pdf]

The major outer sheath protein forms distinct conformers and multimeric complexes in the outer membrane and periplasm of *Treponema denticola.*

Robbins Puthenveetil[1], Sanjiv Kumar[2], Melissa J. Caimano[2,3,4], Abhishek Dey[2#], Arvind Anand[2¥], Olga Vinogradova[5], and Justin D. Radolf [2-4,6,7*]

Departments of [1]Molecular and Cell Biology and [5]Pharmaceutical Sciences, University of Connecticut, Storrs, CT; Departments of [2]Medicine, [3]Pediatrics, [4]Molecular Biology and Biophysics, [6]Genetics and Genome Science, and [7]Immunology, UConn Health, Farmington, CT

[#]Current affiliation: Department of Chemistry, Carnegie Mellon University, Pittsburgh, PA

[¥]Current affiliation: World Sciences Pvt Ltd, Chandigarh, India

Supplementary Figures 1-4.

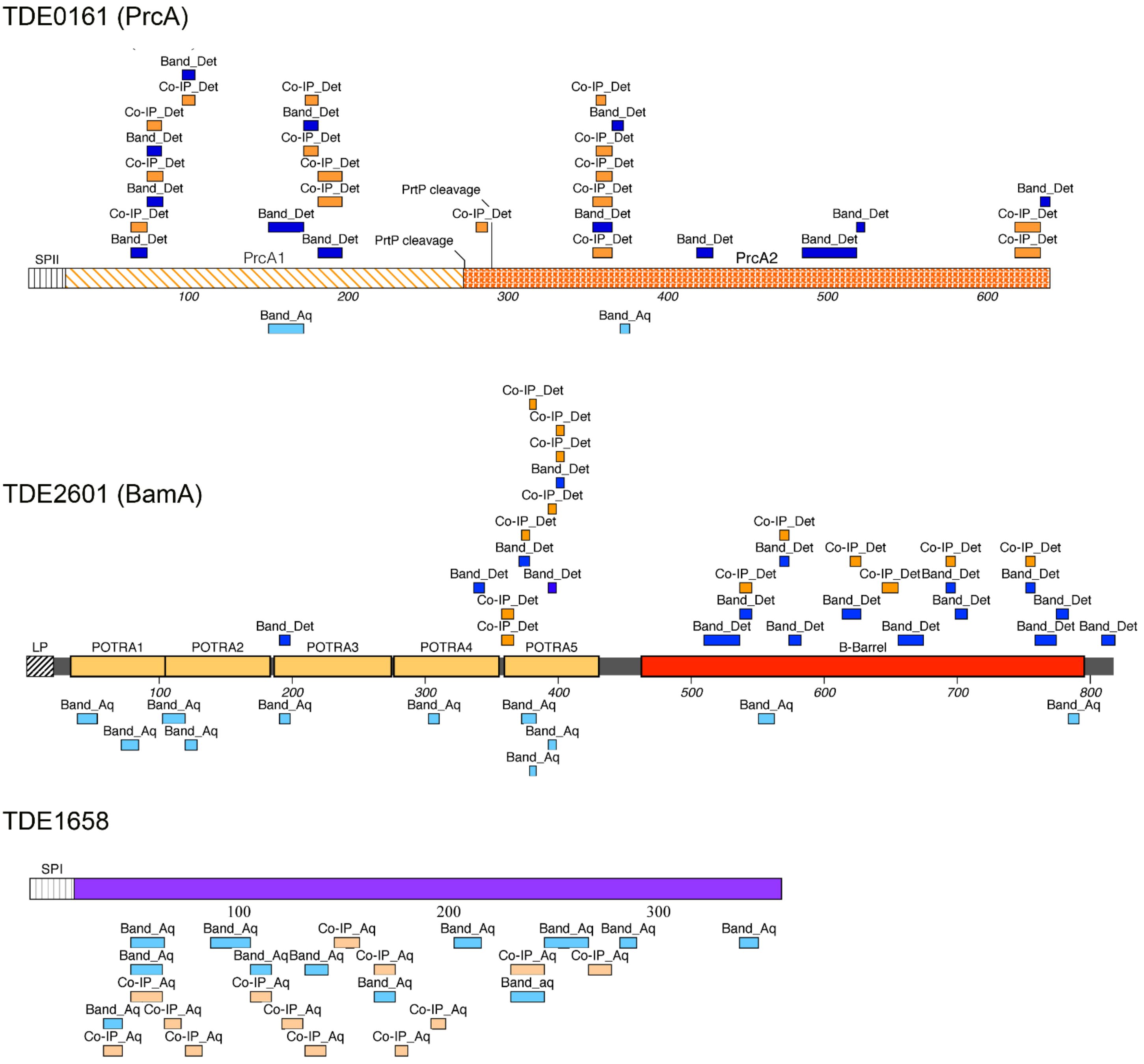

Supplemental Figure 1. Peptides for TDE0161 (PrcA), TDE2601 (BamA), TDE1658 identified by mass spectroscopy analysis of aqueous and detergent phases following either Co-immunoprecipitation (Co-IP_Aq and Co-IP_Det, respectively) or excision of the major MOSP-reactive bands identified BN-PAGE and immunoblot analysis (Band_Aq and Band_Det, respectively). SpI and SpII indicate predicted signal peptides with SPaseI and SpaseII cleavage sites, respectively.

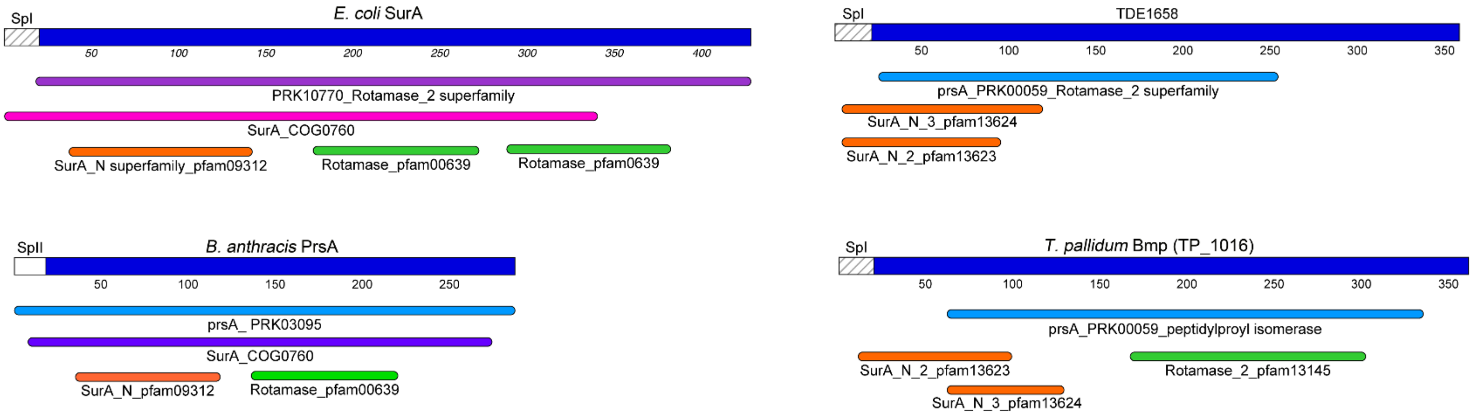

Supplemental Figure 2. Conserved domains in TDE1658, *T. pallidum* TP_1016/Bmp (UniProtKB ID P29725), *Escherichia coli* SurA (UniProtKB ID P0ABZ6) and *Bacillus anthracis* PrsA1 (UniProtKB ID Q81U45) were identified in the NCBI CD database. SpI and SpII indicate predicted signal peptides with SPaseI and SpaseII cleavage sites, respectively.

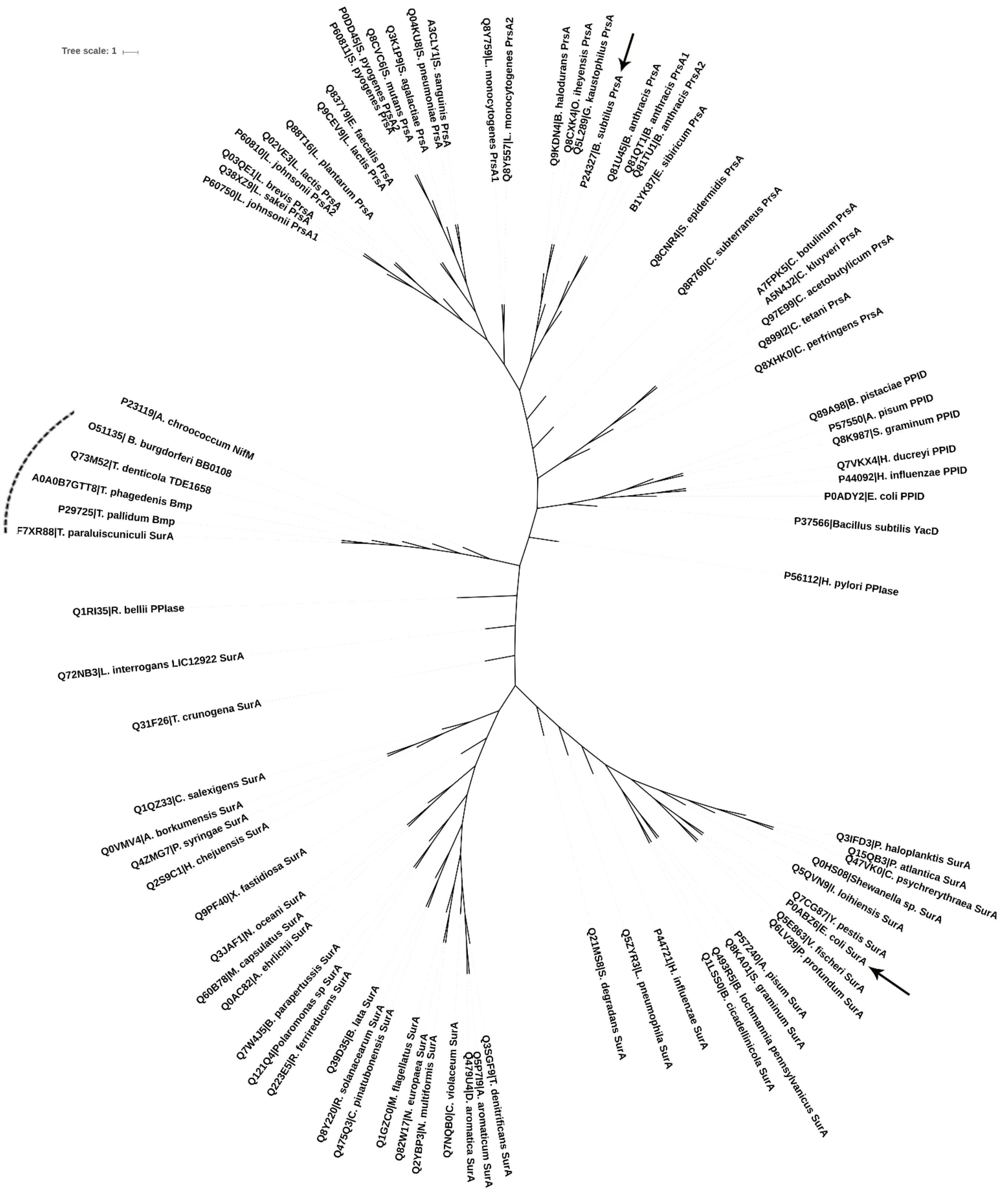

Supplemental Figure 3. Phylogenetic analysis of TDE1658 with representative SurA and PrsA proteins available in the UniProtKB database. Arrows indicate prototypes for SurA (*E. coli* K12) and PrsA (*Bacillus subtilis*). The dashed curve indicates putative spirochetal SurA/PrsA orthologs.

Supplemental Figure 4: Full-length blots used to generate figures containing cropped images.

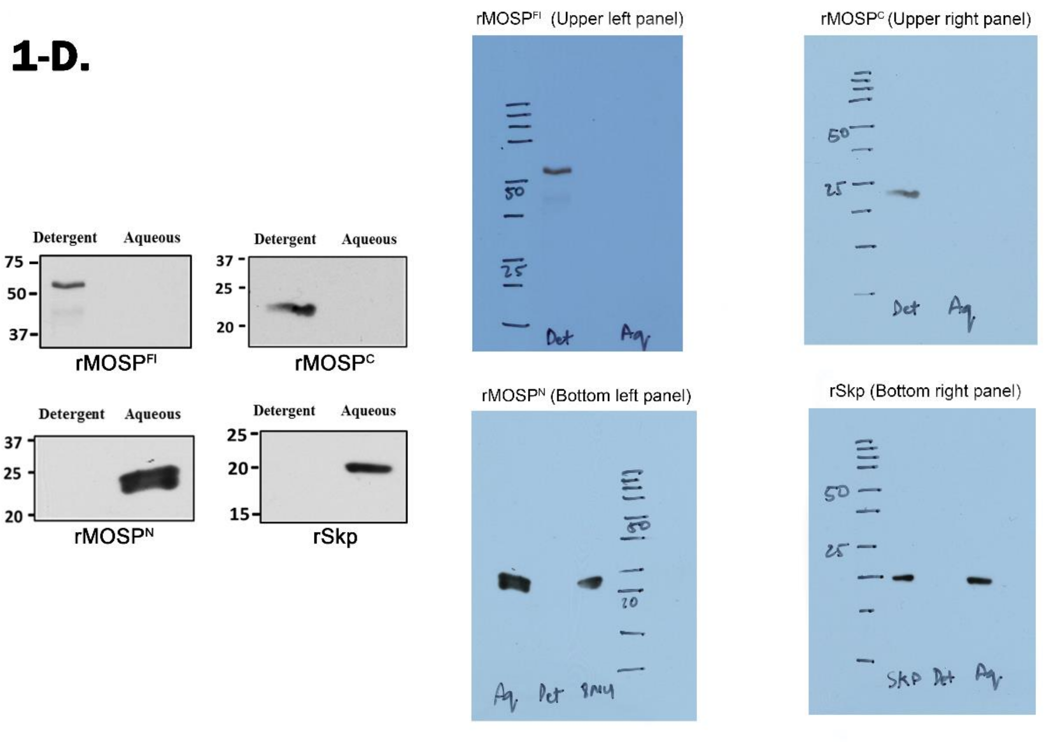

Full length gel images for Figure 1. **(D)** 10 µg of recombinant MOSP^{Fl}, MOSP^{C}, MOSP^{N} and Skp were phase-partitioned in TX-114 and separated on SDS-PAGE. Lanes show detergent-enriched and aqueous phases probed with antisera directed against each recombinant protein.

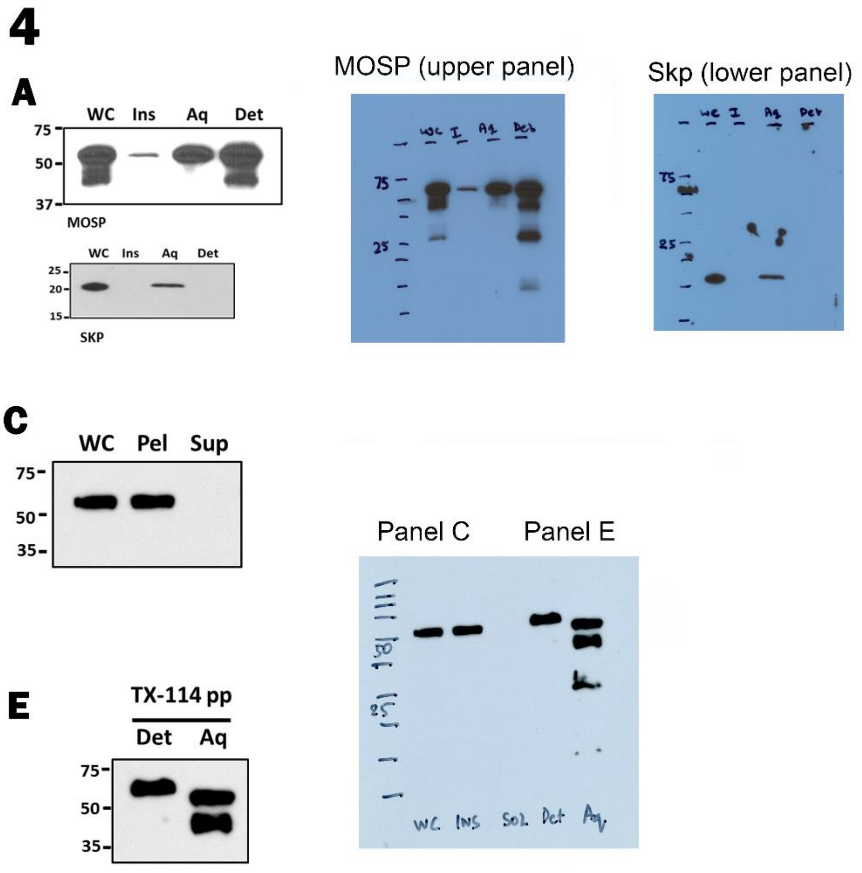

Full length gel images for Figure 4. OM and periplasmic conformers in *T. denticola* are trimeric. (A) Upper panel: detergent-enriched (Det) and aqueous (Aq) phases following TX-114 phase partitioning of *T. denticola*, SDS-PAGE, and immunoblotting with MOSP^{Fl} antiserum; also shown are whole cells (WC) and TX-114-insoluble material (Ins). Lower panel: TX-114 phase partitioning of the periplasmic chaperone Skp in *T. denticola*. **(C)** Immunoblot analysis of the pellet (Pel) and supernatant (Sup) following ultracentrifugation of sonicated *T. denticola* whole cells (WC). **(E)** TX-114 phase partitioning of DDM-solubilized material. Molecular mass standards (kDa) are indicated on the left of each gel.

5-A.

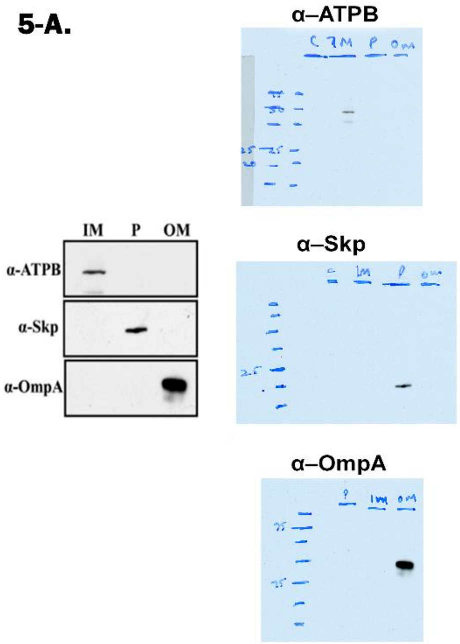

Full length gel images for Figure 5. MOSP expressed in *E. coli* with a PelB signal sequence is bipartite and exclusively OM-localized. (A) Inner membrane (IM), periplasmic (P), and outer membrane (OM) fractions from *E. coli* C41 (DE3) expressing MOSP with PelB signal sequence were separated by SDS-PAGE and immunoblotted against MOSP^{Fl} antiserum. Antisera against ATPB, Skp and OmpA served as markers for the IM, P and OM fractions, respectively.

6-B.

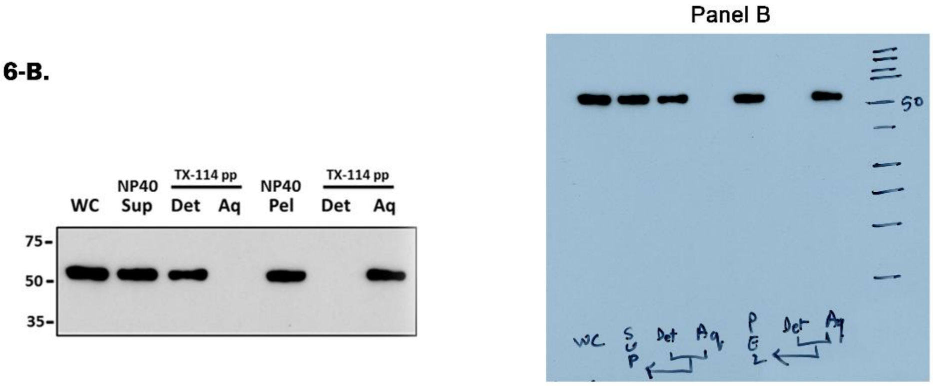

Full length gel images for Figure 6. OM- and periplasmic-MOSP form SDS-stable trimers and distinct multimeric complexes. (B) Immuoblot of the NP-40 soluble (supernatant; NP-40 Sup) and insoluble (pellet; NP40 Pel) fractions obtained by ultracentrifugation which were then separately subjected to TX-114 phase partitioning (TX-114 pp).

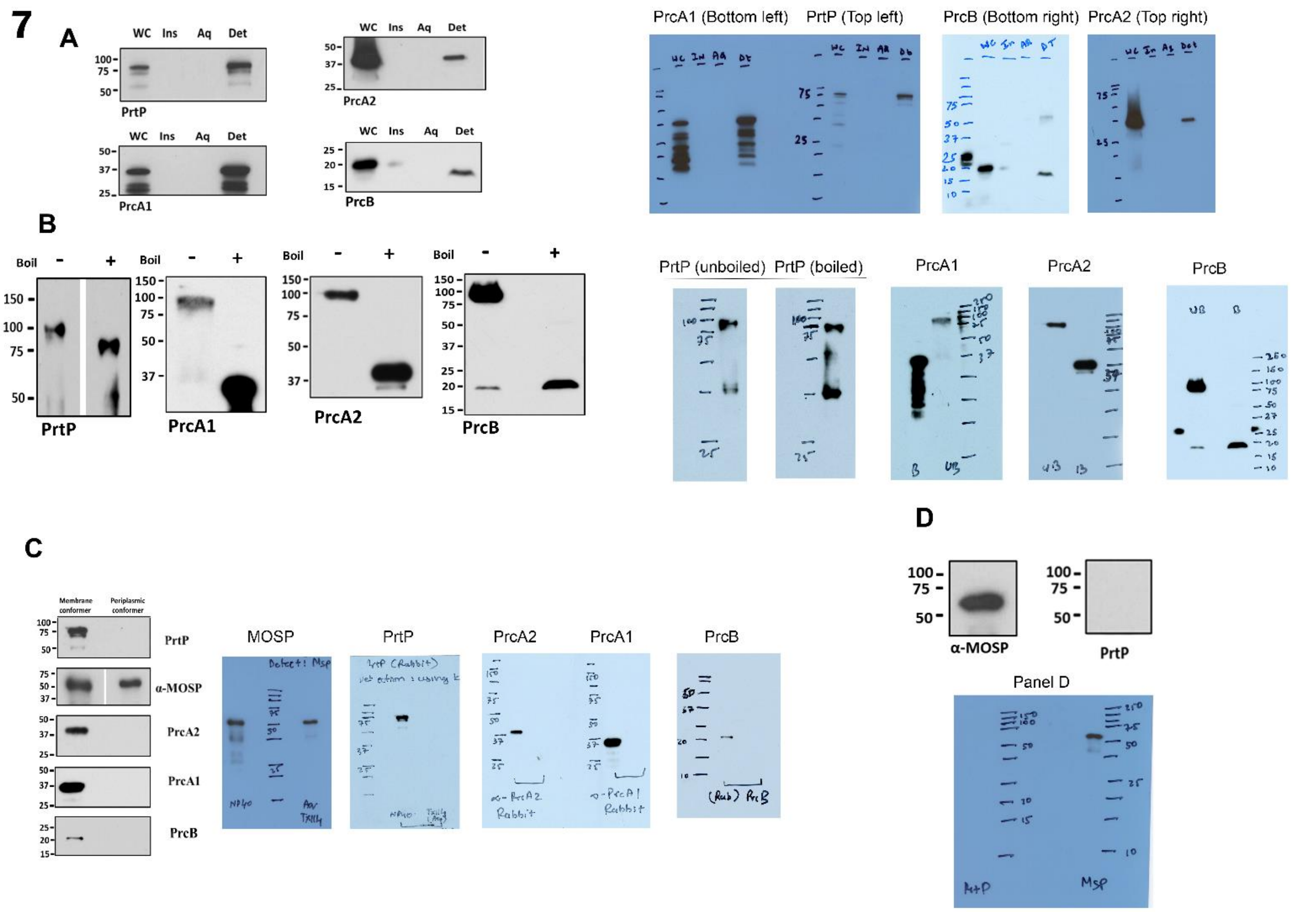

Full length gel images for Figure 7. The dentilisin complex associates only with OM-MOSP. (A) SDS-PAGE and immunoblot analysis of TX-114 phase partitioned *T. denticola* cells using antisera directed against PrtP, PrcA1, PrcA2, and PrcB. Lanes: whole cells (WC), TX-114 insoluble material (Ins), aqueous (Aq) and detergent (Det) phase **(B)** SDS-PAGE gels of the NP-40 supernatant without (**-**) or with (+) boiling followed by immunoblot analysis using the same antisera as in Panel A. **(C)** Eluates from Co-IP of OM and periplasmic conformers were immunoblotted with antisera against the four dentilisin components. **(D)** SDS-PAGE and immunoblot analysis of eluate when 2% DDM was added to the NP-40 supernatant prior to Co-IP with anti-MOSP^{Fl} antiserum. Molecular mass standards (kDa) are indicated on the left of each gel.
